# The interaction of climate, plant, and soil factors drives putative soil fungal pathogen diversity and community structure in dry grasslands

**DOI:** 10.1111/1758-2229.13223

**Published:** 2023-12-20

**Authors:** Shaoyang Zhang, Dandan Fan, Jianshuang Wu, Xianzhou Zhang, Xuliang Zhuang, Weidong Kong

**Affiliations:** ^1^ State Key Laboratory of Tibetan Plateau Earth System, Environment and Resources (TPESER) Institute of Tibetan Plateau Research, Chinese Academy of Sciences Beijing China; ^2^ College of Resources and Environment University of Chinese Academy of Sciences Beijing China; ^3^ Institute of Environment and Sustainable Development in Agriculture Chinese Academy of Agricultural Sciences Beijing China; ^4^ Key Laboratory of Ecosystem Network Observation and Modeling Institute of Geographic Sciences and Natural Resources Research, Chinese Academy of Sciences Beijing China; ^5^ College of Life Sciences Capital Normal University Beijing China

## Abstract

Soil pathogens play important roles in shaping soil microbial diversity and controlling ecosystem functions. Though climate and local environmental factors and their influences on fungal pathogen communities have been examined separately, few studies explore the relative contributions of these factors. This is particularly crucial in eco‐fragile regions, which are more sensitive to environmental changes. Herein we investigated the diversity and community structure of putative soil fungal pathogens in cold and dry grasslands on the Tibetan Plateau, using high‐throughput sequencing. The results showed that steppe soils had the highest diversity of all pathogens and plant pathogens; contrastingly, meadow soils had the highest animal pathogen diversity. Structural equation modelling revealed that climate, plant, and soil had similar levels of influence on putative soil fungal pathogen diversity, with total effects ranging from 52% to 59% (all *p* < 0.001), with precipitation exhibiting a stronger direct effect than plant and soil factors. Putative soil fungal pathogen community structure gradually changed with desert, steppe, and meadow, and was primarily controlled by the interactions of climate, plant, and soil factors rather than by distinct factors individually. This finding contrasts with most studies of soil bacterial and fungal community structure, which generally report dominant roles of individual environmental factors.

## Graphical abstract

- Putative soil fungal pathogen diversity and community structure were explored along a 1200 km grassland transect on the Tibetan Plateau.
- Interactive effect of climate, plant, and soil factors but not individual factors controls pathogen diversity and community structure.
- Pathogen community structure significantly changes along the gradient of desert, steppe and meadow grasslands.

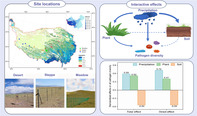

## INTRODUCTION

Soil pathogens play an important role in controlling plant diversity, ecosystem functions, and the health of both humans and animals (Bever et al., 2015; Ciccarone & Rambelli, 2000). They influence plant diversity by altering plant competition and community composition (Delgado‐Baquerizo et al., 2020; Dinoor & Eshed, 1984). Various environmental factors, including regional (precipitation and temperature) and local (plants and soils) factors, have important influences on soil pathogen diversity (Delavaux et al., 2021; Delgado‐Baquerizo et al., 2020). While climate and local environmental factors and their impacts on fungal pathogen communities have been investigated individually, there are limited studies that elucidate the relative contributions of these factors to the community. This is particularly crucial in cold and dry eco‐fragile regions, which are particularly sensitive to climate change and other human disturbances (Zhao et al., 2018).

Fungi and fungus‐like organisms include major soil pathogens, which are generally divided into plant and animal pathogens (Delavaux et al., 2021). Understanding the pathogen diversity, community structure, and their driving environmental factors is critical to predicting the pathogen impacts on human well‐being and ecosystem sustainability under projected climate and land‐use change scenarios. Recent studies have shown that putative pathogen diversity is driven by precipitation (Delavaux et al., 2021; Liu & He, 2021; Spear, 2017), while soil fungal pathogens have also been shown to be sensitive to temperature change (Delgado‐Baquerizo et al., 2020; Marfenina & Danilogorskaya, 2017). On the other hand, soil physical and chemical deterioration could facilitate the growth of soil‐borne pathogens, particularly during grassland degradation and desertification processes (Huber & Haneklaus, 2007). Plants substantially influence soil pathogen community composition by competing for nutrients, while simultaneously providing niches for the growth and proliferation of these pathogens (Bakker et al., 2013; Vandenkoornhuyse et al., 2015). Additionally, the enhanced damage caused by plant pathogens may also increase plant colonization and invasion by animal pathogens (Mendes et al., 2013; Teplitski et al., 2011). These studies have focused on specific environmental factors which control the pathogen diversity and community (Davey et al., 2017; Delavaux et al., 2021; Liu & He, 2021; Pianalto et al., 2018; Singh et al., 2022). However, the assessment of the relative contributions of climate, plant, and soil factors is lacking, which hinders our capacity to predict the responses of soil fungal pathogens to climate change and human disturbances.

We herein investigated the diversity and community structure of putative soil fungal pathogens by dividing them into three groups, all pathogens, plant, and animal pathogens, along a grassland transect across the desert, steppe, and meadow on the Tibetan Plateau. The Tibetan Plateau is more than 4000 meters above sea level, and drought and cold (annual mean temperature < 0°C) substantially limit plant growth (Wang et al., 2018). The alpine grassland ecosystem is particularly eco‐fragile, but has been greening due to rapid climate warming and enhanced precipitation in the past few decades (Zhang et al., 2015; Zhu et al., 2016). To assess how climate, plant, and soil factors control the putative soil fungal pathogen diversity and community structure, we sampled soils along a 1200 km transect covering meadow, steppe, and desert across the Tibetan Plateau. Animal grazing is the typical land use in these grasslands, whose effect on putative soil fungal pathogen diversity was also explored. We hypothesized that (1) putative soil fungal pathogen diversity would substantially differ between meadow, steppe, and desert; (2) interactions of climate, plant, and soil factors would control the diversity of fungal pathogens in a different manner to that of fungal non‐pathogens; and (3) animal grazing would alter the diversity and community composition of fungal pathogens.

## EXPERIMENTAL PROCEDURES

### Field description

The study region is located between 31 and 34 °N latitude and 79 and 93 °E longitude on the plateau, covering a 1200 km east–west transect, and the altitudes range from 4425 m to 4648 m a.s.l. (above sea level). Grasslands, including meadows, steppe, and desert, are the dominant landscape of the Tibetan Plateau, covering 60% of the whole plateau area, approximately 1.5 × 10^6^ km^2^. The plateau is subjected to a monsoon climate in summer, and 90% of the precipitation occurs from July to September (Yang et al., 2014).

In 2006, paired non‐grazed (animal excluding) and grazed treatments (100 m × 100 m each treatment) were established at 12 sites across the plateau (five replicates at 24 treatments, *n* = 120), covering meadow, steppe, and desert grasslands (four sites for each grassland type; Figure S1). Five replicates (1 × 1 m quadrats) were randomly selected within each treatment, and the distance between each quadrat was greater than 30 m. The non‐grazed treatment is adjacent to the grazed treatment at each site, which could minimize the heterogeneity of environmental factors. For the non‐grazed treatment, animals were strictly excluded all year round since 2006. For the grazed treatment, domestic yaks and sheep as well as wild animals were dominant animals, with the grazing intensity range 1.3–2.1, 0.1–0.8, and 0.2–0.4 standard sheep unit (SSU) per hectare for meadow, steppe, and desert grasslands, respectively.

### Soil sampling and plant survey

Soils and plants were sampled in July–August of 2017 at the above‐described 12 sites across the plateau. Five soil cores were sampled (0–20 cm) and mixed into one at each quadrat. Soil samples were sieved by 2 mm to remove roots and stones, and were transported in coolers with ice bags to the ITPCAS laboratory in Beijing (China) and then stored at −80°C for molecular analysis. Within each quadrat, all living plant shoots were collected and stored in envelopes, separated by species, and were dried at 65°C for 48 h to a constant weight. The number of all plant species in each quadrat was used as species richness (*S*). The relative shoot biomass of each plant species (*Pi*/*S*) was used for calculating Pielou evenness (*E*) and the Shannon diversity (*H*), using the following equations:

$$E=\left(-\sum \mathrm{Pi}\;\ln\;\mathrm{Pi}\right)/\ln\;S$$

H=−∑PilnPi

### Soil physicochemical factor analyses and collection of meteorological data

For all soil samples, water content, pH, dissolved organic carbon (DOC), dissolved organic nitrogen (DN), ammonium (NH_4_^+^), and nitrate (NO_3_^−^) were measured. Soil water content (SWC) was determined by oven‐drying soil samples at 105°C to a constant mass (Guo et al., 2015). Soil pH was measured using a pH meter (Sartorius PB‐10, Germany) in a soil‐water slurry (soil: water, 1:2.5). Soil nitrate nitrogen (NO_3_^−^) and ammonium nitrogen (NH_4_^+^) concentrations were measured in 2 M KCl using a Smartchem200 discrete automatic analyser (Alliance, France), and total organic carbon (TOC) was measured using a TOC‐VCPH analyser (TOC‐VCPH, Shimadzu, Japan) (Zhao et al., 2018). Mean temperature and precipitation during the plant growing seasons (referred to as GSAT and GSAP, respectively) were extracted for each sampling site according to the geographic coordinates in ArcGIS 10.2 (Chen et al., 2014).

### DNA extraction and Illumina MiSeq sequencing

Soil DNA was extracted using the DNeasy PowerSoil Pro Kit (QIAGEN, CA, USA), following the manufacturer's instructions. The extracted DNA concentration and purity were measured using a NanoDrop®ND2000 spectrophotometer (NanoDrop Technologies Inc., DE, USA). The fungal ITS2 region was amplified with the forward primer ITS7 (GTGARTCATCGAATCTTTG) (Ihrmark et al., 2012) and the reverse primer ITS4 (TCCTCCGCTTATTGATATGC) (White et al., 1990). This ITS2 region is particularly suitable for short Illumina MiSeq sequencing (Schoch et al., 2012). The PCR mixtures (25 μL) contained 1.0 μM of each primer, 20 ng of the extracted DNA template, 12.5 μL of Phusion Mastermix with HF Buffer, and 10.5 μL of ddH_2_O (Thermo Fisher Scientific, USA). The PCR amplification was conducted with an initial denaturation at 95°C (3 min), followed by 30 cycles of denaturation at 94°C (1 min), annealing at 55°C (1 min), and extension at 72°C (1 min), with a final extension at 72°C (10 min). The purified PCR products were accurately quantified using Qubit4.0 pooled at equal molar amounts and sequenced on the Illumina MiSeq PE 250 platform at Magigene Technologies Co., Ltd. (Guangzhou, China). The fungal raw sequencing reads have been deposited in the NCBI sequence reads archive with project ID PRJNA922344.

### Bioinformatics analysis

QIIME2 v2021.2 was used for the high throughput sequence quality control and construction of the feature table and taxonomic classification at the amplicon sequence variants (ASV) level (Bolyen et al., 2019). The dada2 plugin was used to trim and truncate poor regions of ITS raw reads and remove chimeras (Telatin, 2021). The truncation and trimming were set to p‐trim‐left 0, ‐p‐trunc‐len 210. All ASVs with <5 reads were removed to eliminate possible PCR/sequencing artefacts. Taxonomic analysis was carried out using the QIIME2 feature‐classifier plugin (Bokulich et al., 2018) and taxonomic assignments were made using the UNITE fungal database (v8.3) (Nilsson et al., 2019). We compared the resulting taxonomic identities against the FUNGuild database (Nguyen et al., 2016; Tanunchai et al., 2023), which can identify putative fungal pathogens and the non‐pathogens (Delavaux et al., 2021). Putative soil fungal pathogens were identified by FUNGuild and included both exclusively pathogenic taxa and those with mixed lifestyles, with confidence levels of either highly probable or probable (Delgado‐Baquerizo et al., 2020). To further verify the FUNGuild results, another independent database, FungalTraits (version 1.2) was also used to identify the putative soil fungal pathogens (Põlme et al., 2021).

### Statistical analyses

All statistical analyses were carried out on three pathogen groups: all, plant, and animal pathogens. The relative abundance of pathogens was defined as the proportion of the ITS sequences. The diversity function in the vegan software package was used to estimate the diversity by calculating richness (ASV number), Shannon diversity, and Pielou's evenness in R 4.0.1. The richness of putative soil fungal pathogens was referred to as the ASV number (Margalef, 1968). The Shannon diversity of putative soil fungal pathogens was calculated from the relative abundance of all pathogen ASVs (Shannon & Weaver, 1963). The evenness of putative soil fungal pathogens indicates how evenly different pathogens are distributed in the community (Simpson, 1949). SPSS 25.0 (IBM Inc., Armonk, NY) was used to conduct one‐way ANOVA to assess the significance of differences in abundance, Shannon diversity, and richness of plant and animal pathogens under different treatments and grassland types. Relationships between fungal diversity and environmental factors were examined with linear or quadratic regressions using Sigmaplot 14.0 (Systat Software Inc., San Jose, CA, USA). Generalized linear mixed effect modelling (GLM) of SPSS 25.0 (IBM Inc., Armonk, NY) was used to assess the impact of grassland type, temperature, and precipitation on pathogen Shannon diversity and richness (Delavaux et al., 2021). Structural equation modelling (SEM) used the partial least‐squares path modelling (PLS‐PM) algorithm to explore the direct, indirect, and interactive effects of all measured variables on pathogen diversity using the R package plspm (v 0.4.7) (Tenenhaus et al., 2005). PLS‐PM is widely applied to explain and predict relationships in multivariate datasets (Cui et al., 2016; Wagg et al., 2014). SEM incorporated all the environmental factors, including climate (growing season accumulated precipitation and temperature) and plant (biomass, plant diversity, plant richness) factors, soil physicochemical properties (pH, DN, DOC, TOC), and pathogen diversity indices (the diversity and richness of all pathogens, plant pathogens and animal pathogens). Indirect effects are defined as multiple pathway coefficients between predictor and response variables including all possible pathways excluding the direct effect. The model incorporated all environmental factors, except those with loading values less than 0.7, such as NO_3_^−^ and NH_4_^+^. The total effect is the sum of direct effects and indirect effects. The final model was chosen from all constructed models based on the goodness of fit (GOF) statistic, a measure of the model's overall predictive power. The same SEM method was also applied to the non‐pathogen diversity.

Nonmetric multidimensional scaling (NMDS) is an unconstrained ordination approach, which produces an ordination based on distance or dissimilarity matrix. Here NMDS was used to visualize the community dissimilarity of putative soil fungal pathogens. Bray–Curtis dissimilarity was calculated from the pathogen ASV relative abundance using the ‘vegdist’ function in the vegan package, which was used in the NMDS. Two‐way PERMANOVA using Bray–Curtis dissimilarity was performed with the vegan ‘adonis’ function to quantify the different significance of putative pathogen communities. Redundancy Analysis (RDA) is a method to extract and summarize the variation in a set of response variables (e.g., pathogen community structure) that can be explained by a set of environmental factors. Therefore, RDA was performed to explore significant relationships between the pathogen communities and environmental factors using the package vegan in R (Segata et al., 2011). Variation partitioning analysis (VPA) was performed to determine the contribution of soil, climate, and plant factors by partitioning the effect of co‐correlated factors on the pathogen community variation using R (Wu et al., 2017). VPA analysis of the non‐pathogen community was also carried out, and the results were compared with the pathogen community.

## RESULTS

### General ASV assignment and number across databases

A total of 4070 fungal ASVs were assigned to functional guilds using the FUNGuild database. ASVs of putative fungal pathogens accounted for 12.7% of the fungal ASVs, among which 1154 ASVs represented plant pathogens and 672 ASVs were animal pathogens. The independent database Fungaltrait generated 4561 fungal ASVs, of which 776 ASVs and 144 ASVs were for plant and animal pathogens, respectively (Figure S2). The FungalTraits database achieved 12% more assignments to fungal ASVs than the FUNGuild database in the current study. The FUNGuild database showed that the relative abundance of putative plant pathogens ranged from 8.14% to 46.4% of all fungal sequences, and animal pathogens stayed between 3.31% and 39.59% (Figure S3). The putative soil fungal pathogen community composition was dominated by *Alternaria*, *Gibberella*, and *Acremonium*, whose relative abundances (percentage of the fungal sequences) were 3.33%, 2.43%, and 0.74%, respectively. Meadow soils showed a significantly higher relative abundance of all pathogens than steppe and desert soils, and grazing did not influence the relative abundance of all pathogens (Figure S4). Similar results were also observed for the relative abundance of the most common plant and animal pathogens (Figure S5).

### Soil fungal pathogen diversity

**FIGURE 1:**
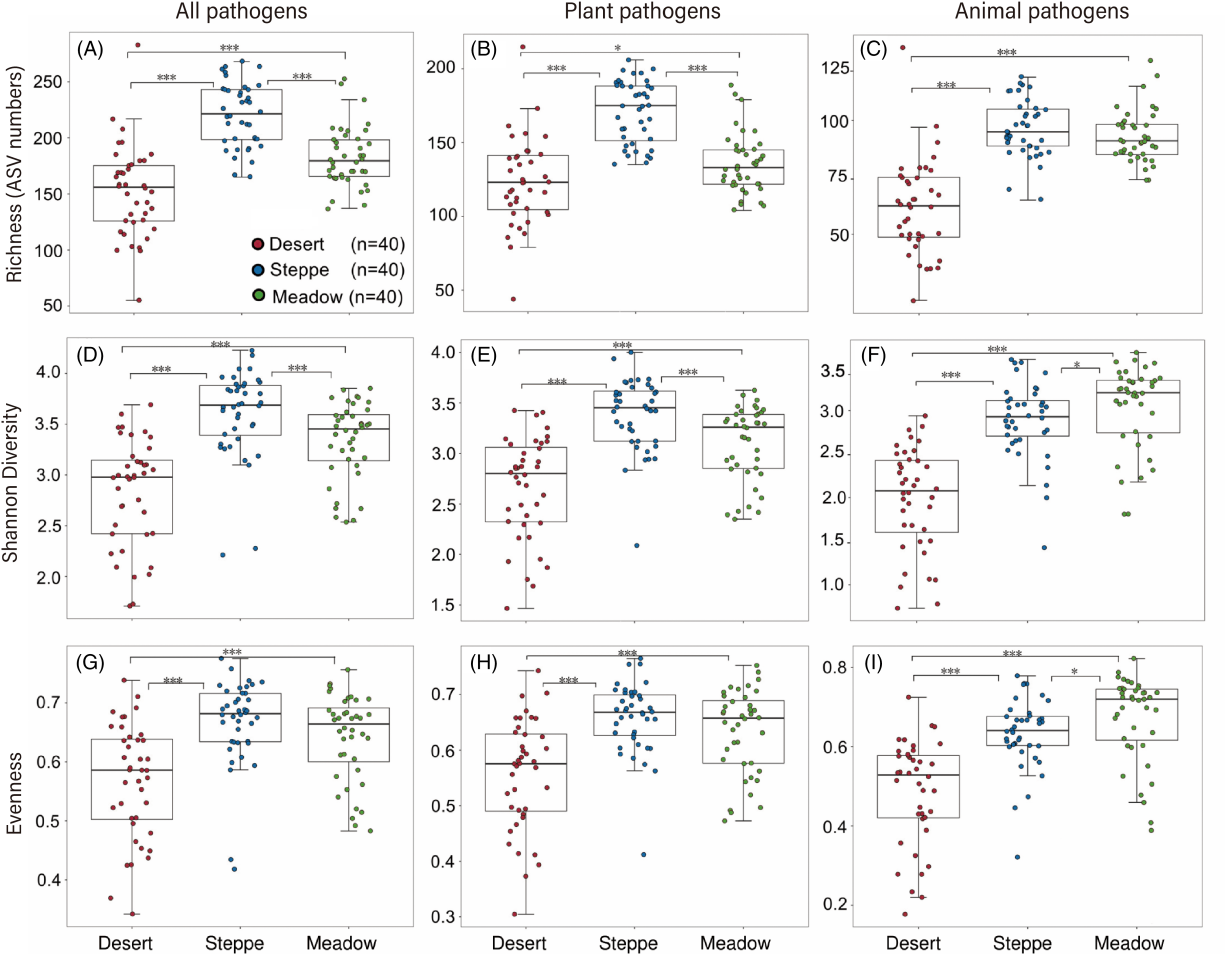
The richness, Shannon diversity, and evenness of all pathogens (A–C), plant pathogens (D–F), and animal pathogens (G–H) in desert, steppe, and meadow soils. The symbols on the horizontal lines indicate the different significance levels of the effects (*p < 0.05, **p < 0.01, ***p < 0.001).

The grassland type had strong effects on pathogen diversity (richness, Shannon diversity, and evenness; *p* < 0.001, Figure 1). The richness of all pathogens significantly differed among the desert, steppe, and meadow soils (*p* < 0.001). In particular, all pathogen richness in steppe soils was higher than that in desert and meadow soils by 44.7% and 20.6%, respectively. Similar to the richness, the highest values of both Shannon diversity and evenness for all pathogens were observed in steppe soils (all *p* < 0.05). The plant pathogen diversity (richness, Shannon diversity, and evenness) showed a similar pattern as all pathogens across the three grassland types. In contrast, the highest animal pathogen Shannon diversity and evenness occurred in meadow soils, which was significantly higher than in steppe and desert soils (all *p* < 0.05). Grazing did not significantly influence the richness, Shannon diversity, and evenness in desert, steppe, and meadow soils for all the putative pathogens (all *p* > 0.05; Figures S6–S8). GLM results further validated that grassland type significantly influenced the putative pathogen diversity (richness, Shannon diversity; all *p* < 0.01), but neither grazing nor the interaction between grazing and grassland type influenced the diversity (all *p* > 0.05; Table S1).

### The interaction of environmental factors controlling pathogen diversity

**FIGURE 2:**
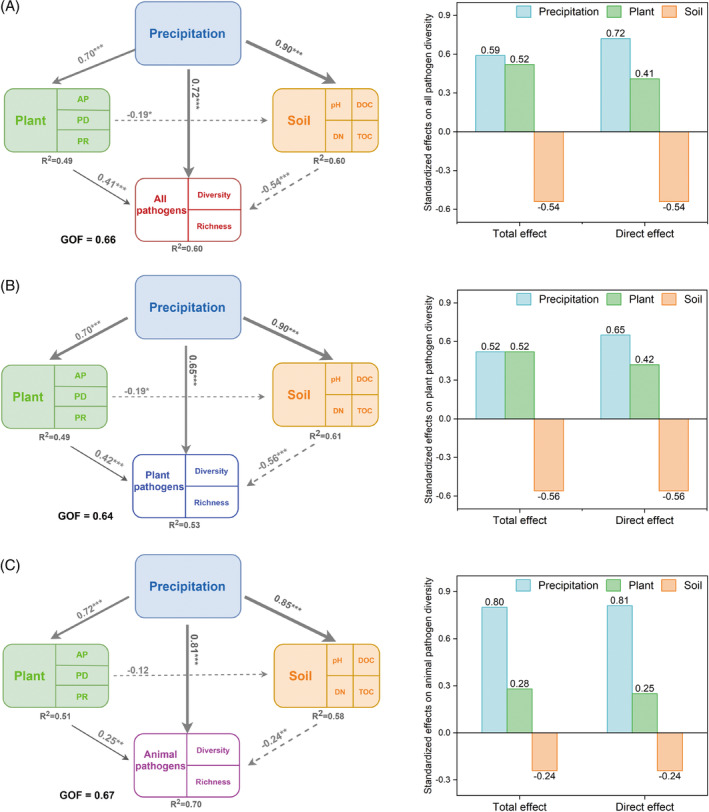
Direct, indirect, and standardized effects of climate, plant, and soil factors on the diversity of all pathogens (A), plant pathogens (B), and animal pathogens (C) in grassland soils by structural equation modelling. Numbers adjacent to arrows indicate different significance levels of the effects (*p < 0.05, **p < 0.01, ***p < 0.001). Arrow width is proportional to the relative strength of path coefficients. Dashed and solid lines indicate negative and positive correlations, respectively. AP, aboveground plant‐biomass; DN, dissolved nitrogen; DOC, dissolved organic carbon; PD, plant diversity; PR, plant richness; TOC, total organic carbon. The model was analysed using the goodness of fit (GOF) statistic. *R* ^2^, coefficient of determination. The model deletes NO_3_^−^ and NH_4_^+^, because their loading values are less than 0.7.

Structural equation modelling (SEM) was used to identify the direct and indirect relationships between pathogen diversity (all pathogens, plant, and animal pathogens) and environmental factors (precipitation, plant, and soil factors). For all pathogens, the total standardized effects of precipitation, plant, and soil factors on pathogen diversity were 0.59, 0.52, and −0.54 (Figure 2), respectively. In contrast, precipitation exhibited a substantially stronger direct effect (direct standardized effect 0.72) on all pathogen diversity than plant (0.41) or soil factors (−0.54). Precipitation, therefore, enhanced pathogen diversity both directly and indirectly by influencing plant and soil factors (all *p* < 0.001). Plant factors, including aboveground plant biomass, diversity, and richness, positively influenced the diversity of all pathogens. In contrast, soil factors, the interaction of nutrients and pH, negatively influenced diversity. The effects of climate, plant, and soil factors on plant pathogen diversity considered individually were similar to all pathogens. For animal pathogen diversity, precipitation exhibited a more dominant role (0.81) than all pathogens and plant pathogens in both direct and indirect ways, while plant (0.25) and soil (0.24) factors exhibited weaker effects (all *p* < 0.001). SEM analysis was also applied to the non‐pathogen diversity, and the results showed that the total standardized effects of precipitation, plant, and soil factors were 0.58, 0.43, and −0.62 (Figure S9), respectively. Precipitation exhibited a substantially stronger direct effect (direct standardized effect 0.82) on the non‐pathogen diversity than plant (0.33) and soil factors (−0.62).

### Correlations between soil fungal pathogen diversity and environmental factors

Correlations between putative soil fungal pathogen diversity and climate, plant, and soil factors were calculated using linear or quadratic regression regressions. The pathogen diversity was significantly correlated with climate (growing season accumulated precipitation, GSAT; growing season average temperature, GSAT; and aridity), plant (plant richness and plant diversity), and soil (DN, DOC, and pH) factors (Figures S10–S12). All pathogen richness was significantly correlated with precipitation in quadratic regression (*R* ^2^ = 0.469, *p* < 0.001), and negatively correlated with temperature and aridity (both *p* < 0.001). All pathogen Shannon diversity and evenness increased with increasing precipitation but decreased with increasing temperature (all *p* < 0.001; Figure S10). Soil DN, DOC, and pH all showed significant relationships with all pathogen richness and Shannon diversity (all *p* < 0.05; Figure S11). In contrast, soil pH values for the highest animal pathogen richness (7.3 vs. 7.6) and Shannon diversity (6.9 vs. 7.4) were slightly lower than those in plant pathogens. All pathogen diversity was significantly positively correlated with plant diversity (*R* ^2^ = 0.382, *p* < 0.001) and plant richness (*R* ^2^ = 0.375, *p* < 0.001), but was not significantly correlated with plant evenness (Figure S12). The diversity of plant and animal pathogens exhibited similar regression patterns to those of all pathogens. Correlations between precipitation and soil factors (pH, DOC, DN, TOC) were calculated using linear regression models. The results showed that precipitation was negatively correlated with soil pH (*R* ^2^ = 0.702, *p* < 0.001), and positively correlated with DOC, DN, and TOC (all *p* < 0.001; Figure S13).

### Pathogen community structure and controlling factors

**FIGURE 3:**
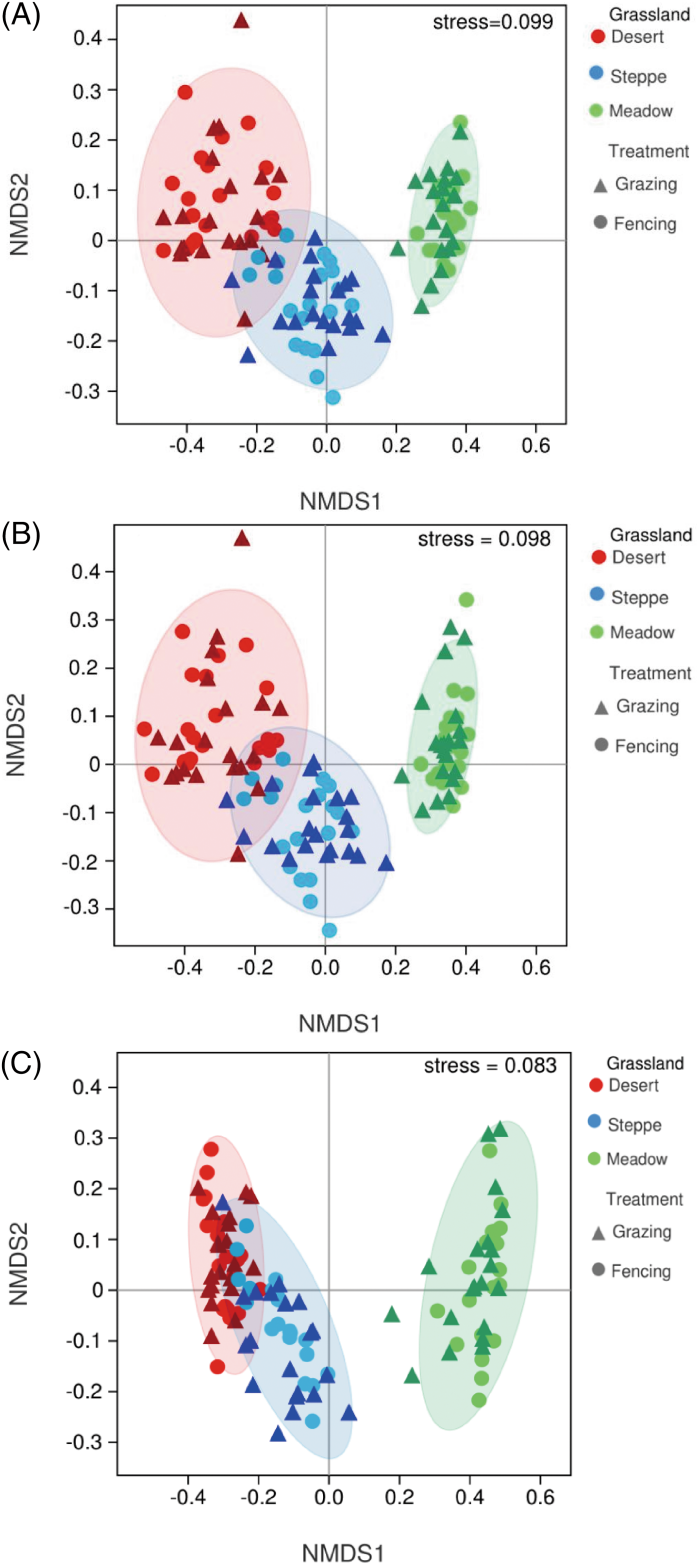
Soil fungal pathogen community structure based on nonmetric multidimensional scaling (NMDS) in grasslands. (A–C) show all pathogens, plant pathogens, and animal pathogens, respectively.

**FIGURE 4:**
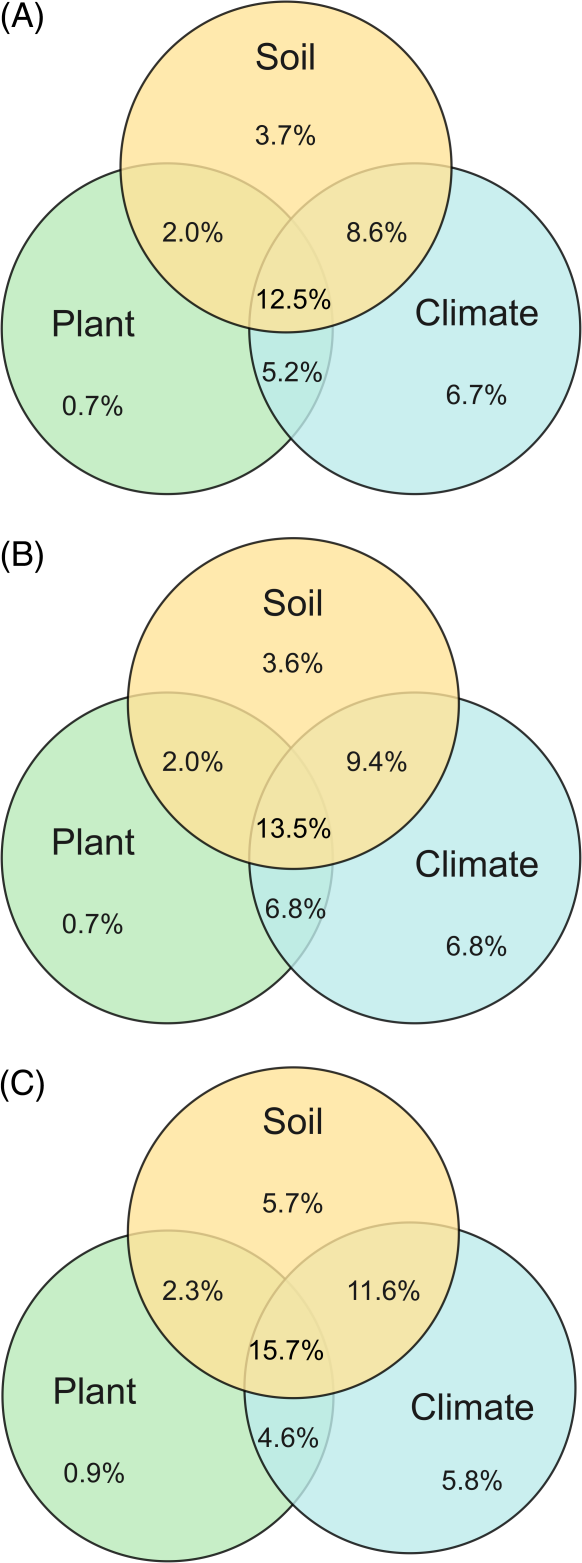
Variation partitioning analysis on the relative contributions of climate, plant, and soil factors to community structure of all pathogens (A), plant pathogens (B), and animal pathogens (C). Soil: pH, DOC, DN, NH_4_^+^, NO_3_^−^, TOC; plant: plant diversity, plant richness, aboveground plant‐biomass; Climate: aridity degree, growing season average temperature, growing season accumulated precipitation. DOC, dissolved organic carbon; DN, dissolved nitrogen; NH_4_^+^, ammonium; NO_3_^−^, nitrate; TOC, total organic carbon.

Non‐metric multidimensional scaling (NMDS) was performed to elucidate the differences in soil fungal pathogen community structure based on the Bray–Curtis dissimilarity and phylogenetically weighted UniFrac distance matrixes. The NMDS results revealed that the communities of all pathogens and plant pathogens significantly changed along the gradient of desert, steppe, and meadow grasslands, but were less influenced by grazing (Figure 3). Similar results were also observed from two‐way PERMANOVA and Adonis (Table S2). Redundancy analysis (RDA) was used to further examine the changes in pathogen community structures and their relationships with climate, plant, and soil factors. Community structures were collectively driven by all environmental factors, among which precipitation, soil pH, and plant richness played dominant roles (Figure S14 and Table S3). The joint and separate contributions of these environmental factors were quantified using variation partitioning analysis (VPA). VPA results showed that these environmental factors jointly explained 39.3% of all pathogen community variation (Figure 4). In particular, the interactions of environmental factors explained more variation (28.3%) than the separate contributions of climate (6.7%), soil (3.7%) and plant (0.7%) factors. Similar results were observed in the plant and animal pathogen communities. For non‐pathogen community structure, the interactions of environmental factors explained more variation (13.7%) than the separate contributions of climate (6.1%), soil (3.6%), and plant (0.8%) factors (Figure S15).

## DISCUSSION

### The interaction of environmental factors controls putative soil fungal pathogen diversity

Our results demonstrated that climate, plant, and soil factors had similar total effects on putative soil fungal pathogen diversity, ranging from 52% to 59% (Figure 2), highlighting the key role of interactions between environmental factors in driving diversity. This finding is consistent with observations that putative soil pathogen fungal richness is dependent on the interaction between precipitation and temperature (Delavaux et al., 2021) and that plant pathogen richness is weakly correlated with climate factors after incorporating all environmental factors (Makiola et al., 2022). Plant and soil factors commonly change dramatically in concert with climate factors (Ladron de Guevara & Maestre, 2022), and their interactive effects on soil microbes are thus overlooked (Orwin et al., 2016). This confirmation of the importance of interactions contrasted with conclusions commonly drawn in studies of the diversity of soil bacteria and fungi, which generally conclude that diversity is dominated by specific environmental factors such as precipitation, soil factors such as pH, or plant factors (Bossio et al., 1998; Hawkes et al., 2011; Hedlund et al., 2003). Our results showed that the total effects of climate, plant, and soil factors on all pathogen diversity (0.59, 0.52, −0.54) were more distinct than those on non‐pathogen diversity (0.58, 0.43, −0.62). This difference in total effects indicates that the strong interactive effects of environmental factors may be a distinct driver of putative soil fungal pathogen diversity. Overall, our findings support the hypothesis that interactions between environmental factors are more important than the influence of individual environmental factors in controlling putative soil pathogen fungal diversity (Dionne et al., 2007).

Among the environmental factors, precipitation showed the strongest direct effect on putative soil fungal pathogen diversity in the Tibetan Plateau grasslands. The RDA and regression models provided robust support for precipitation being the best predictor of pathogen diversity (Figure S14), consistent with previous studies that precipitation is the key driver of soil pathogen diversity (Delavaux et al., 2021; Makiola et al., 2022). Precipitation directly enhances plant biomass and diversity, and regulates soil pH and nutrients (Figure S13), which collectively drive soil pathogen diversity (Chen et al., 2021; Yang et al., 2022). Additionally, increased precipitation reduces the aggregation of generalist pathogens and facilitates the movement and distribution of pathogens in soils, subsequently increasing pathogen diversity (Strengbom et al., 2006). In contrast, temperature exhibited a substantially weaker influence on putative soil fungal pathogen diversity than did precipitation based on the RDA (Table S3), although warming has been shown in other studies to directly affect fungal pathogen diversity (Liu & He, 2021).

Our data also confirmed that plant and soil factors exerted strong effects on putative soil fungal pathogen diversity. Pathogens are pervasive and abundant, leaving no plant species or ecosystems free of their influence (Bever et al., 2015). Our findings are consistent with that diverse plant communities sustain high pathogen diversity (Makiola et al., 2022; Rottstock et al., 2014), although the strength of this relationship depends on the pathogen and plant species. Pathogen diversity was also influenced by soil nutrients and pH (Figure 2 and S11). Our results showed that soil carbon and nitrogen were significantly positively correlated with pathogen diversity, consistent with observations that fertilization or unbalanced nutrient supply, especially nitrogen, often benefit pathogens (Hartmann et al., 2014). The nutrient‐enhancing effect on pathogen diversity may be a result of increased nutrient supply by reducing plant reliance on arbuscular mycorrhizal fungi, which can suppress pathogens (Lekberg et al., 2021; Veresoglou & Rillig, 2012). Our results demonstrated that the interaction of soil factors negatively influenced the pathogen diversity, this could be caused by soil pH. Soil pH was negatively correlated with pathogen diversity, and the pH‐suppressing effect was especially pronounced in alkaline conditions. This is in agreement with observations that increased soil pH usually decreases soil pathogens, including *Fusarium oxysporum*, *Ralstonia solanacearum*, and *Rhizoctonia solani* (Liu et al., 2019; Shen et al., 2018). Additionally, soil pH could indirectly influence soil pathogen diversity and community composition by changing the soil microbial community (Li et al., 2019; Liu et al., 2023).

In the current study, plant pathogen diversity was highest in steppe soils, while animal pathogen diversity was highest in meadow soils. This was supportive of the finding that lower pH increases the richness and abundance of soil pathogens (Shen et al., 2018), consistent with the highest plant pathogen diversity in steppe soils under alkaline conditions (Figures S11 and S16). Plant life history strategy has been proposed to play a major influence on plant pathogen diversity (Garcia‐Guzman & Heil, 2014). Plants with rapid growth are more susceptible to pathogens which usually results in high pathogen diversity (Makiola et al., 2019). Some generalist animal pathogens can infect both plants and animals (Singh et al., 2022), thus animal pathogen diversity can also have a strong response to plant factors. Our results showed that obligate animal pathogens dominated the grassland soils compared to generalist pathogens (Figure S5). Consistent with the high animal pathogen diversity, it was observed that enhanced precipitation can facilitate the movement of animal pathogens, further increasing pathogen diversity (Milici et al., 2020).

### The interaction of environmental factors controls soil fungal pathogen community structure

Pathogens are integral ecosystem agents that play vital roles in soil nutrient cycling, plant nutrition, and pathology (Bever et al., 2015). Our results demonstrated that the interaction among climate, soil, and plant factors was substantially more important than individual environmental factors in controlling the putative soil fungal pathogen community structure (Figure 4). Furthermore, the interactive contribution of all environmental factors was also much greater than that of separate environmental factors to the non‐pathogen community structure (Figure S15). This contrasts with previous studies that climate, plant, or soil factors have strong separate influences on pathogen community structure (Makiola et al., 2022; Mukhi et al., 2020). This is likely due to the lack of a wide range of environmental factors, which hinders the understanding of the relationships between environments and pathogens (Makiola et al., 2022). Our results demonstrated that the pathogen community structure differed significantly with grassland type (Figure 2), and the community structure was also most strongly influenced by climate factors (Figure 4). Climate factors could be particularly important in explaining pathogen community structure because they represent an external driver of all other environmental factors (Yamaura et al., 2011). Plant and soil factors are partly determined by climate suitability (Makiola et al., 2022). Under the community ecological framework, plants are regarded as resource patches for soil microbes, including pathogens, and individual environmental factors as local filters that drive pathogen community structure (Borer et al., 2016). Therefore, our findings highlight the interactive effects of these environmental factors on pathogen community structure, which has been largely ignored.

Putative soil fungal pathogens assigned in this study were mainly affiliated with the genera *Alternaria*, *Gibberella*, and *Coniochaeta* (Figure S3). Previous studies showed that the relative abundance of these fungal pathogens was higher in tropical coastal regions than in the continental interior, indicating that precipitation and temperature were the key factors affecting pathogen community composition (Delgado‐Baquerizo et al., 2020; Singh et al., 2022). Members of the genus *Alternaria* are the most widely distributed pathogens in global soils, causing diseases in a wide range of economically important plants, including wheat, sunflower seeds, tomatoes, apples, and olives (Meena et al., 2016). *Gibberella* and *Coniochaeta* are commonly associated with wheat, rice, barley, rye, and mixed feed (Desjardins, 2003), and can even attack the human and animal digestive tracts and circulatory systems (Mueller et al., 2012; Sutton et al., 2009). These pathogens cause a potential health risk when contaminated grass is grazed by livestock because some generalist pathogens can infect both plants and animals. Such negative impacts of pathogens may be further enhanced by climate change (Kilpatrick & Randolph, 2012; Singh et al., 2022; Zhou et al., 2019). Tibetan grasslands potentially host a pathogen bank, so understanding of the pathogen community and mitigation of its effects is particularly important for human health on the Tibetan Plateau. Our results support the hypothesis that pathogen communities are driven by the interaction of climate, plant, and soil factors in Tibetan Plateau grassland soils. Taxonomic and functional group assignments rely on well‐informed reference databases, which may be lacking particularly in Tibetan Plateau soils. Different pathogen databases could generate differences in the definition of the same pathogens. For example, the genus *Fusarium* was assigned to ‘plant pathogen’ in FungalTraits, while FUNGuild assigned *Fusarium* to ‘Animal Pathogen‐Endophyte‐Lichen Parasite‐Plant Pathogen‐Soil Saprotroph‐Wood Saprotroph’ with the confidence level ‘possible’. In the current study, the results generated from the pathogen database FUNGuild were generally consistent with those from the other pathogen database FungalTraits.

### Pathogen diversity and community composition responses to grazing

Our results showed that grazing exhibited little effect on soil fungal pathogen diversity and compositions (Figure S5), which is in disagreement with our hypothesis. The little effects likely result from that pathogen diversity and community structure were dominated by the interaction of climate, plant, and soil factors rather than by individual environmental factors. In a recent study, we observed that grazing substantially decreased plant biomass/diversity and soil‐dissolved organic carbon (Fan et al., 2023). Therefore, the little grazing effects on pathogen diversity indicate that grazing did not influence the interactive effects of environmental factors on the pathogen community. Furthermore, these results collectively highlight the dominant role of interactions of environmental factors on soil fungal pathogen diversity and community structure. The little effect contrasted with a previous study that grazing significantly altered pathogen compositions in plant roots (Delavaux et al., 2021). This may result from the different grazing periods, because the grazing period was 11 years in our study, while the other was 20–50 years. Additionally, low grazing intensity frequently does not influence native plant species, which consequently did not alter pathogen compositions due to the host‐specificity of soil pathogens (Bever et al., 2015).

## CONCLUSIONS

In this study, we comprehensively characterized putative soil fungal pathogen diversity and community structure and their driving factors in the cold and dry grasslands of the Tibetan Plateau. We found that the interactions between climate, plant, and soil factors were the most important drivers of putative soil fungal pathogen diversity and community structure in these grasslands, contrasting with the findings of many studies of the diversity of non‐pathogenic soil bacteria and fungi. The strongest direct effects on the putative soil fungal pathogen diversity were associated with climate factors relative to plant and soil factors. Our findings provide novel insights into the factors driving soil pathogen diversity and increase understanding of the sensitivity of soil pathogens to environmental change in grassland ecosystems. Furthermore, these pathogens potentially pose risks to the health of humans and animals, and this risk could be further exacerbated by rapid climate change on the Tibetan Plateau. This may be a particularly urgent challenge on the plateau, with the native human population less resistant to certain serious pathogens, such as *Mycobacterium tuberculosis* (Li et al., 2012).

## AUTHOR CONTRIBUTIONS

**Shaoyang Zhang:** Data curation (lead); formal analysis (lead); investigation (lead); writing – original draft (lead). **Dandan Fan:** Data curation (supporting). **Jianshuang Wu:** Investigation (supporting); resources (supporting); writing – review and editing (supporting). **Xuliang Zhuang:** Writing – review and editing (equal). **Weidong Kong:** Conceptualization (lead); funding acquisition (lead); project administration (lead); resources (lead); supervision (lead); writing – original draft (lead); writing – review and editing (lead).

## CONFLICT OF INTEREST STATEMENT

The authors declare that there is no conflict of interest.

## Supporting information


**Data S1.** Supporting Information

## Data Availability

The raw sequencing reads generated have been deposited in the NCBI Sequence Read Archive under project ID PRJNA922344.
