# Supplementary material for: The interaction of climate, plant, and soil factors drives putative soil fungal pathogen diversity and community structure in dry grasslands
Source: Environ Microbiol Rep. 2023 Dec 20;16(1):e13223. doi: 10.1111/1758-2229.13223 (PMC10866062; doi:10.1111/1758-2229.13223)
Supplement: Supplementary file 1 — Data S1. Supporting Information [file EMI4-16-e13223-s001.docx]

**SUPPLEMENTARY FIGURES**

**Figure S1** Site locations in deserts, steppes, and meadows along climate gradients on the northern Tibetan Plateau (n = 120). Different colors represent different grasslands. The grazed plot at each site was chosen randomly within 1-2 km of the fenced (ungrazed) plot. Grazing and fenced plots could not be distinguished on the map. At each sampling site, five quadrats (1 m × 1 m) were set up randomly within a flat area of 200 m × 200 m in both grazed and fenced plots.

**
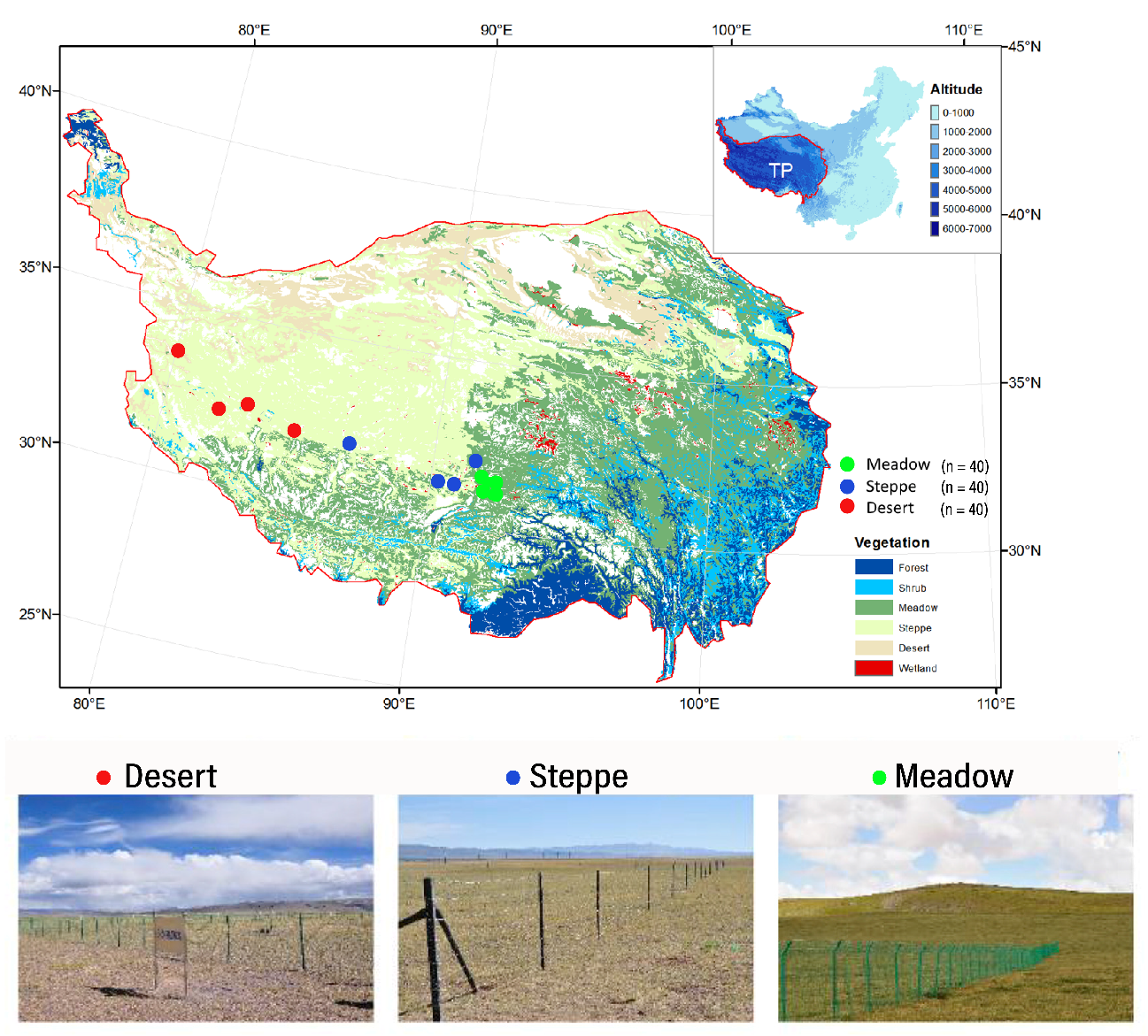
**

**Figure S2** The number of fungal pathogen ASVs identified by the database FUNGuild and database FungalTraits.
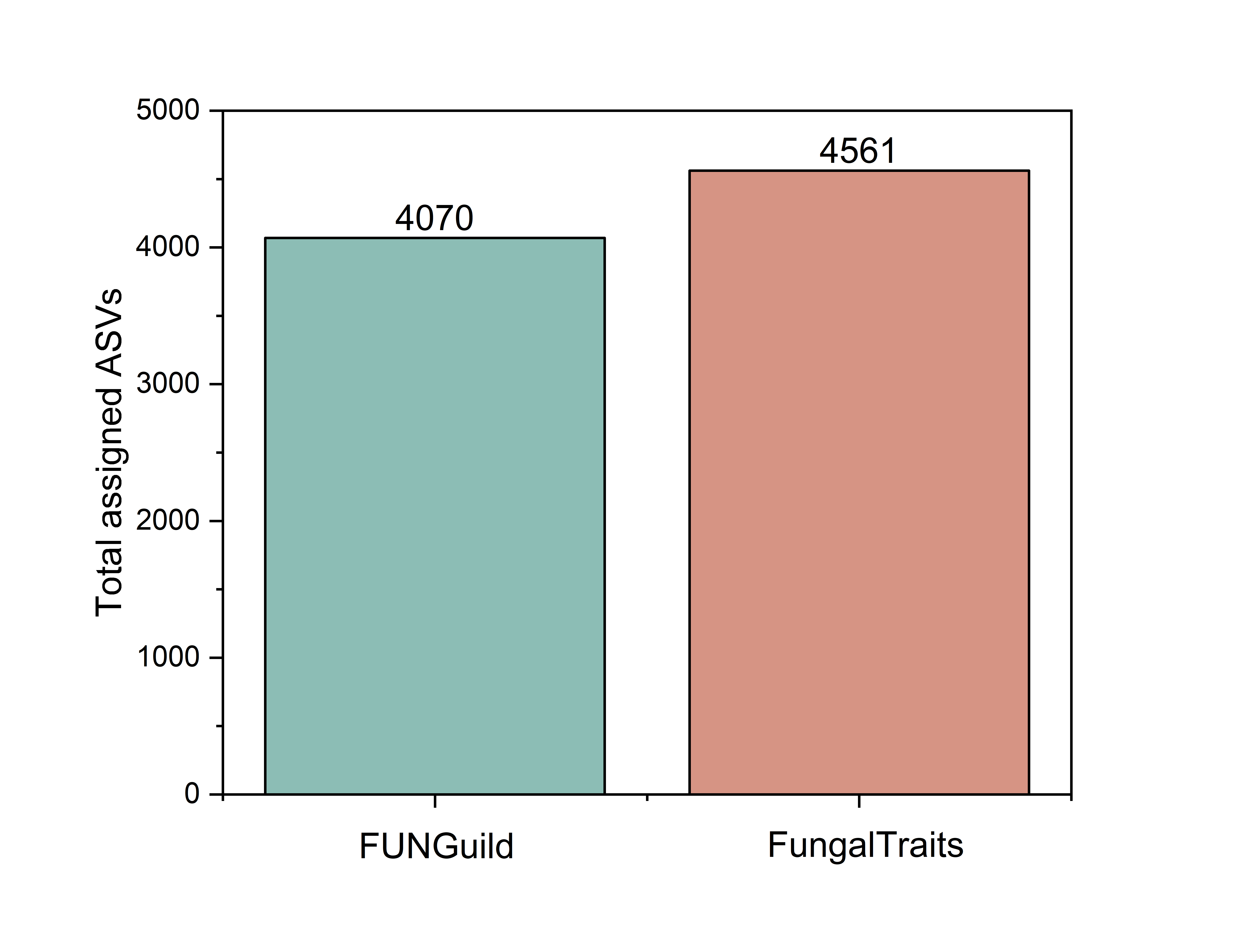


**Figure S3** Relative abundance of potential plant and animal pathogens in the Tibetan Plateau. **(a**, **c**) Distribution of the relative abundance (percentage of the ITS sequences) of plant pathogens and animal pathogens in each sample. (**b**, **d**) Relative abundance (percentage of the ITS sequences) of the most common plant pathogens and animal pathogens identified.


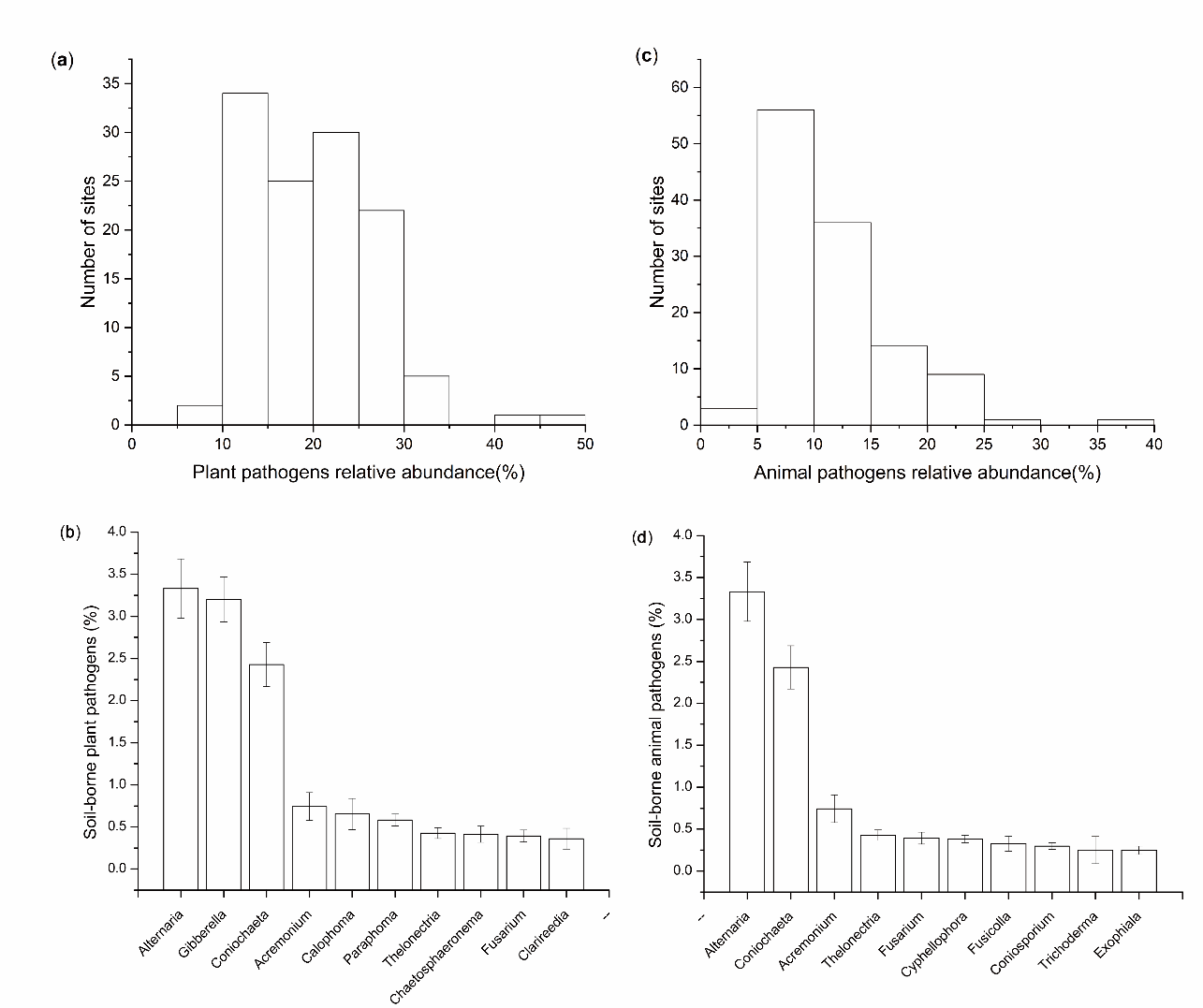


**Figure S4** Identity of potential plant and animal pathogens Tibetan. Mean values (± s.e.) for the relative abundance (percentage of the ITS sequences) of potential (a) all pathogens, (b) plant pathogens, and (c) animal pathogens across grasslands and fencing.


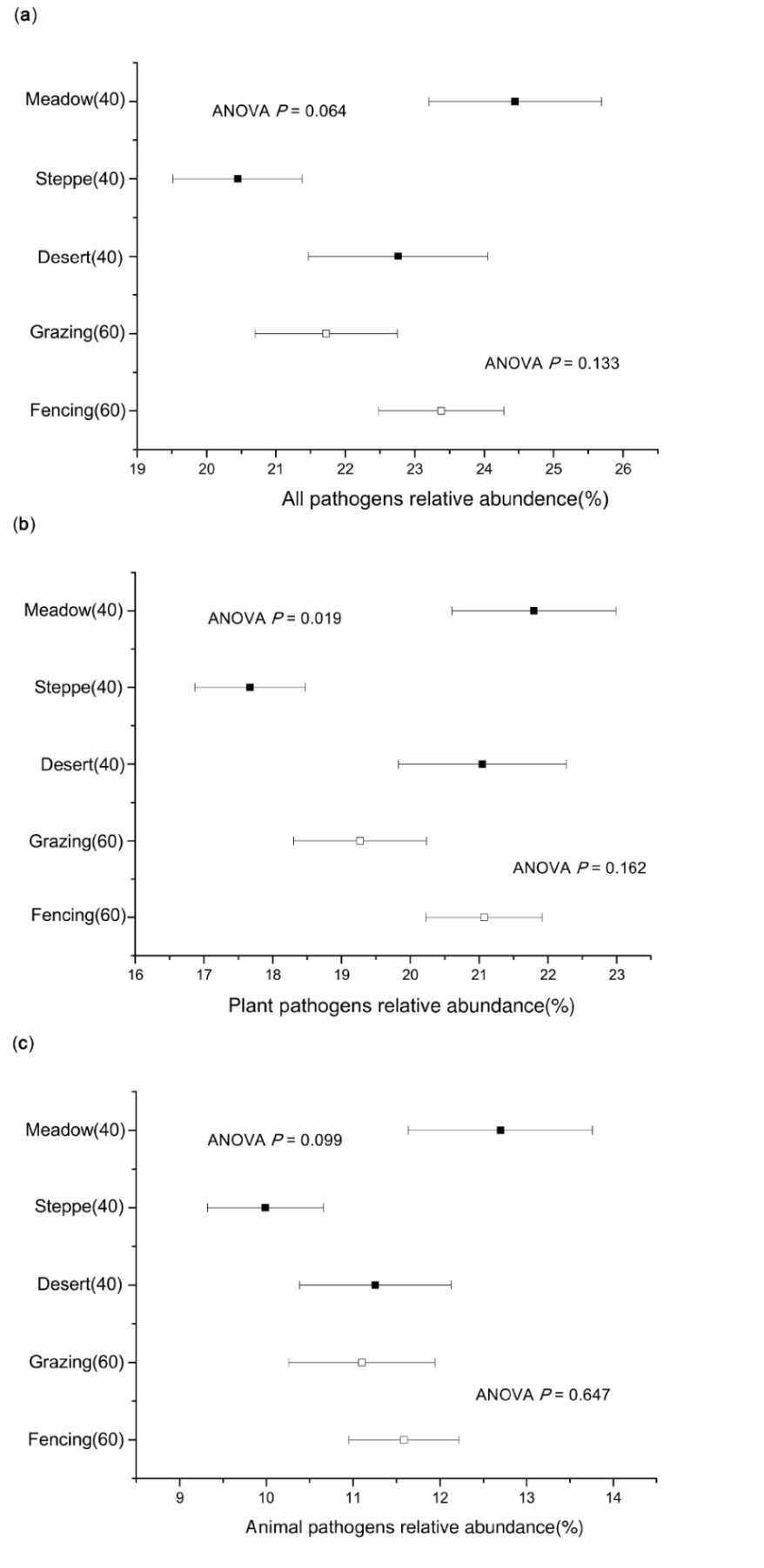


**Figure S5** Relative abundance (percentage of the ITS sequences) of the most common (a) plant pathogens and (c) animal pathogens identified in fencing and grazing (mean ± s.e.). Relative abundance (percentage of the ITS sequences) of the most common (b) plant and (d) animal pathogens identified in desert, steppe, and meadow (mean ± s.e.).


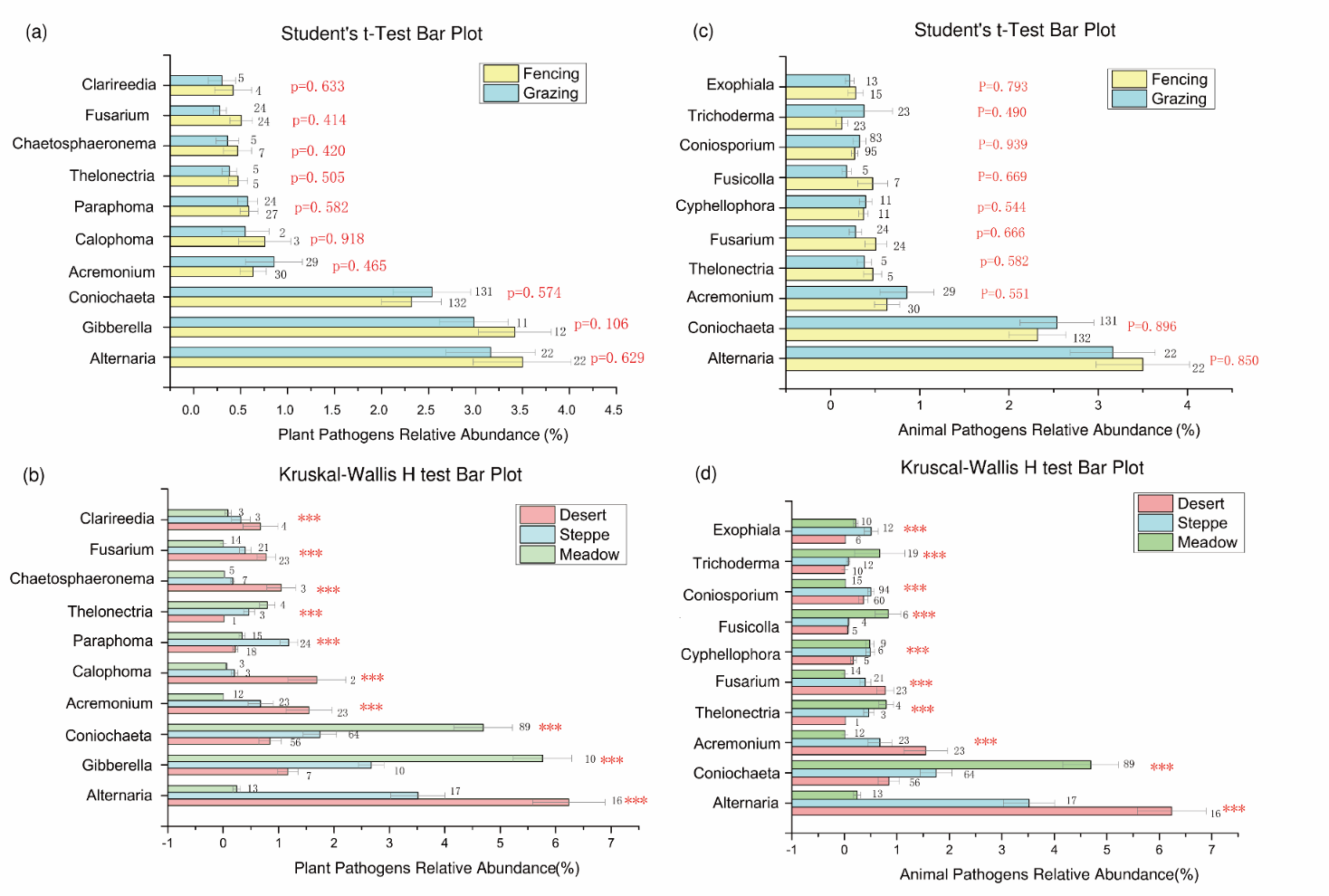


**Figure S6** The diversity of all pathogens includes diversity (a), richness (b), and evenness (c) in fencing and grazing. DF: fenced desert; DG: grazed desert; SF: fenced steppe; SG: grazed steppe; MF: fenced meadow; MG: grazed meadow.


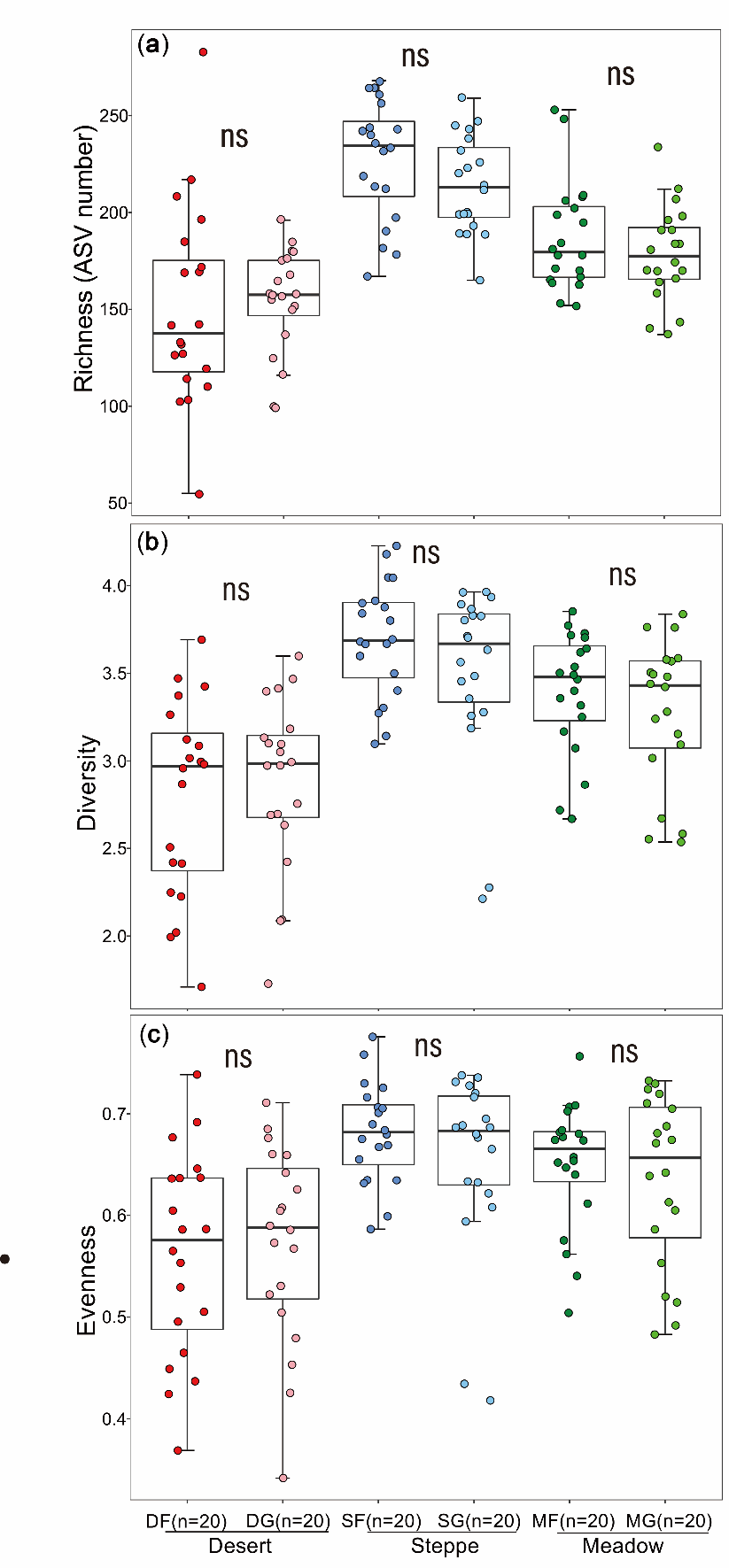


**Figure S7** The diversity of plant pathogens includes diversity (a), richness (b), and evenness (c) in fencing and grazing. DF: fenced desert; DG: grazed desert; SF: fenced steppe; SG: grazed steppe; MF: fenced meadow; MG: grazed meadow.

**
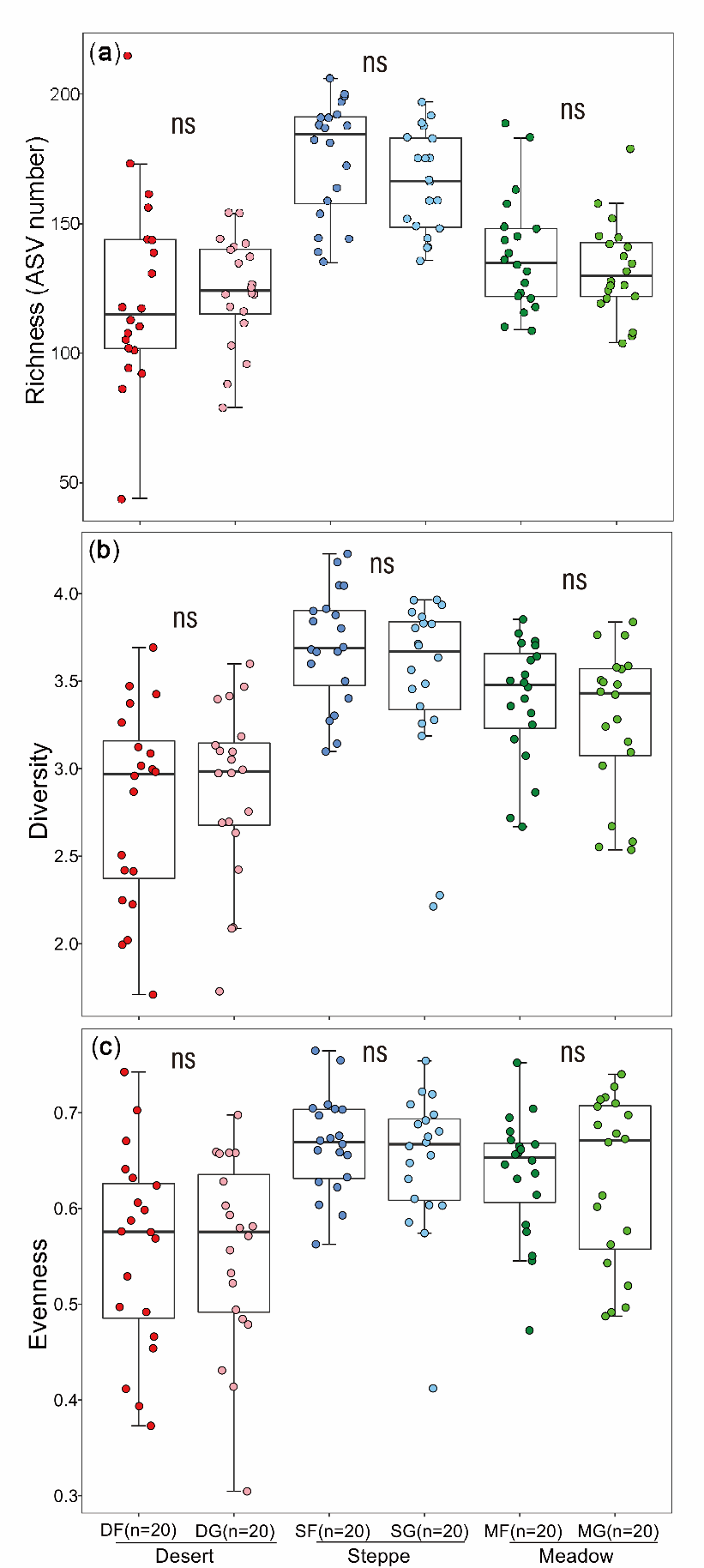
**

**Figure S8** The diversity of animal pathogens includes diversity (a), richness (b), and evenness (c) in fenced and grazed grasslands. DF: fenced desert; DG: grazed desert; SF: fenced steppe; SG: grazed steppe; MF: fenced meadow; MG: grazed meadow.

**
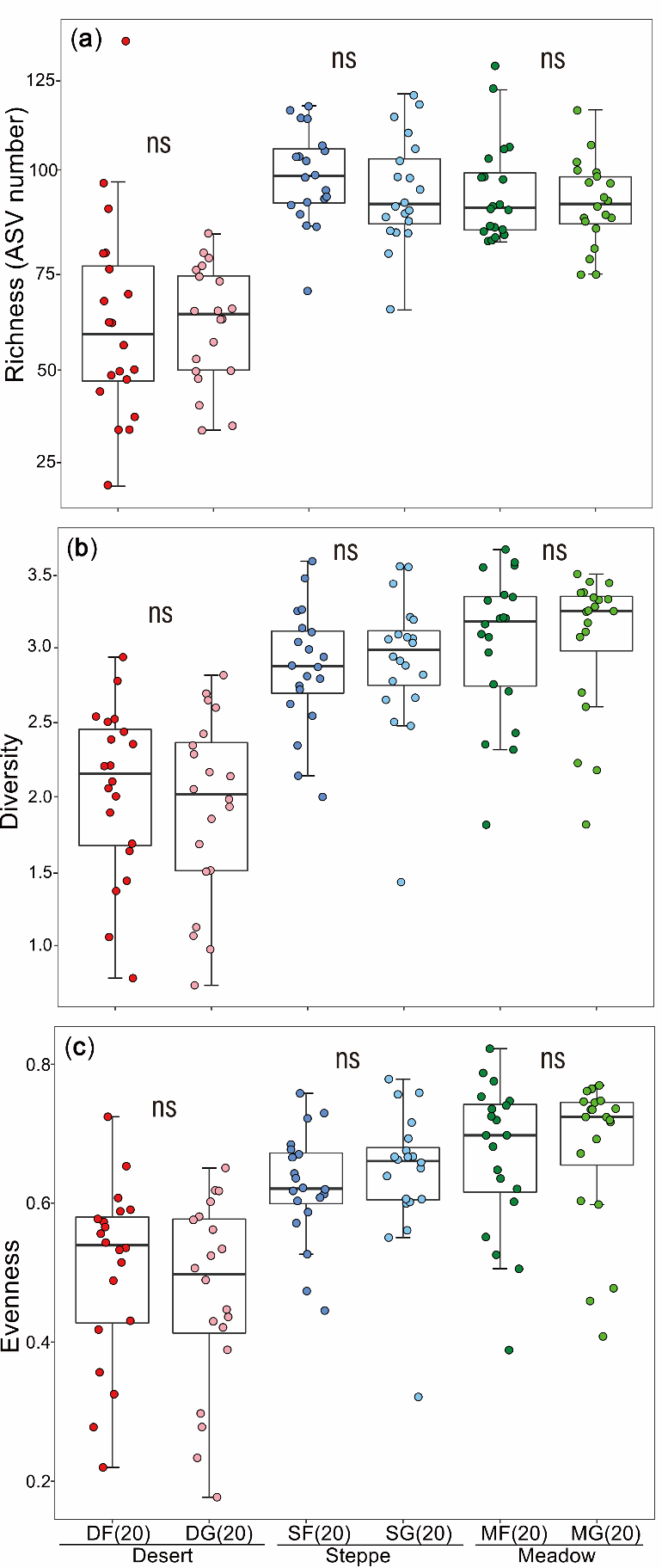
**

**Figure S9** Direct, indirect, and standardized effects of climate, plant, and soil factors on non-pathogen diversity by structural equation modeling. Numbers adjacent to arrows indicate different significance levels of the effects (* < 0.05, ** < 0.01, *** < 0.001). Arrow width is proportional to the relative strength of path coefficients. Dashed and solid lines indicate negative and positive correlations, respectively. AP: aboveground plant-biomass; PD: plant diversity; PR: plant richness; DOC: dissolved organic carbon; DN: dissolved nitrogen; TOC: total organic carbon. The model was analyzed using goodness of fit (GOF) statistics. R^2^, coefficient of determination. The model deletes NO_3_^-^ and NH_4_^+^ whose loading value is less than 0.7.

**
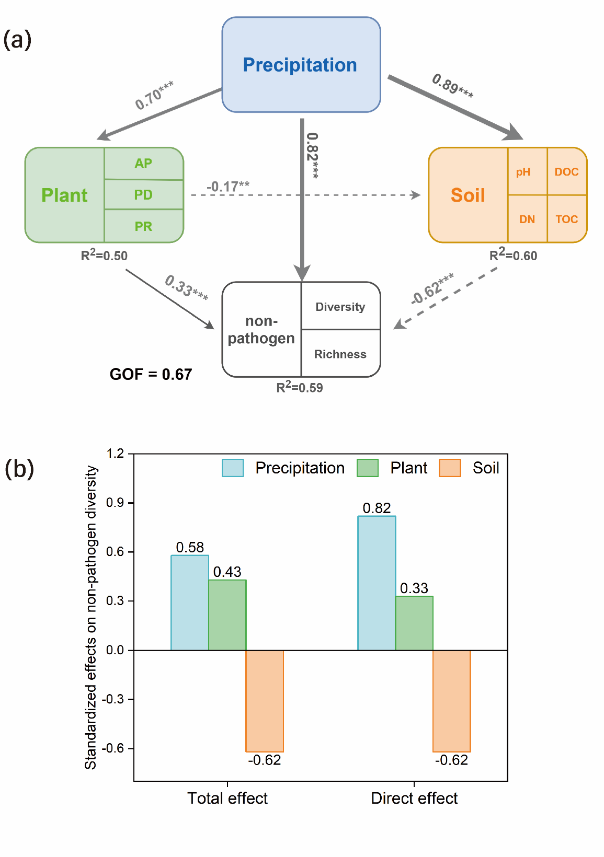
**

**Figure S10** Correlations between diversity of various types of pathogens and climate factors. Regressions of pathogen diversity (richness, Shannon diversity, and evenness) with GSAP (a-c), GSAT (d-f), and aridity (g-i), including all pathogen diversity, plant pathogen diversity, and animal pathogen diversity. GSAP: growing season accumulated precipitation; GSAT: growing season average temperature.


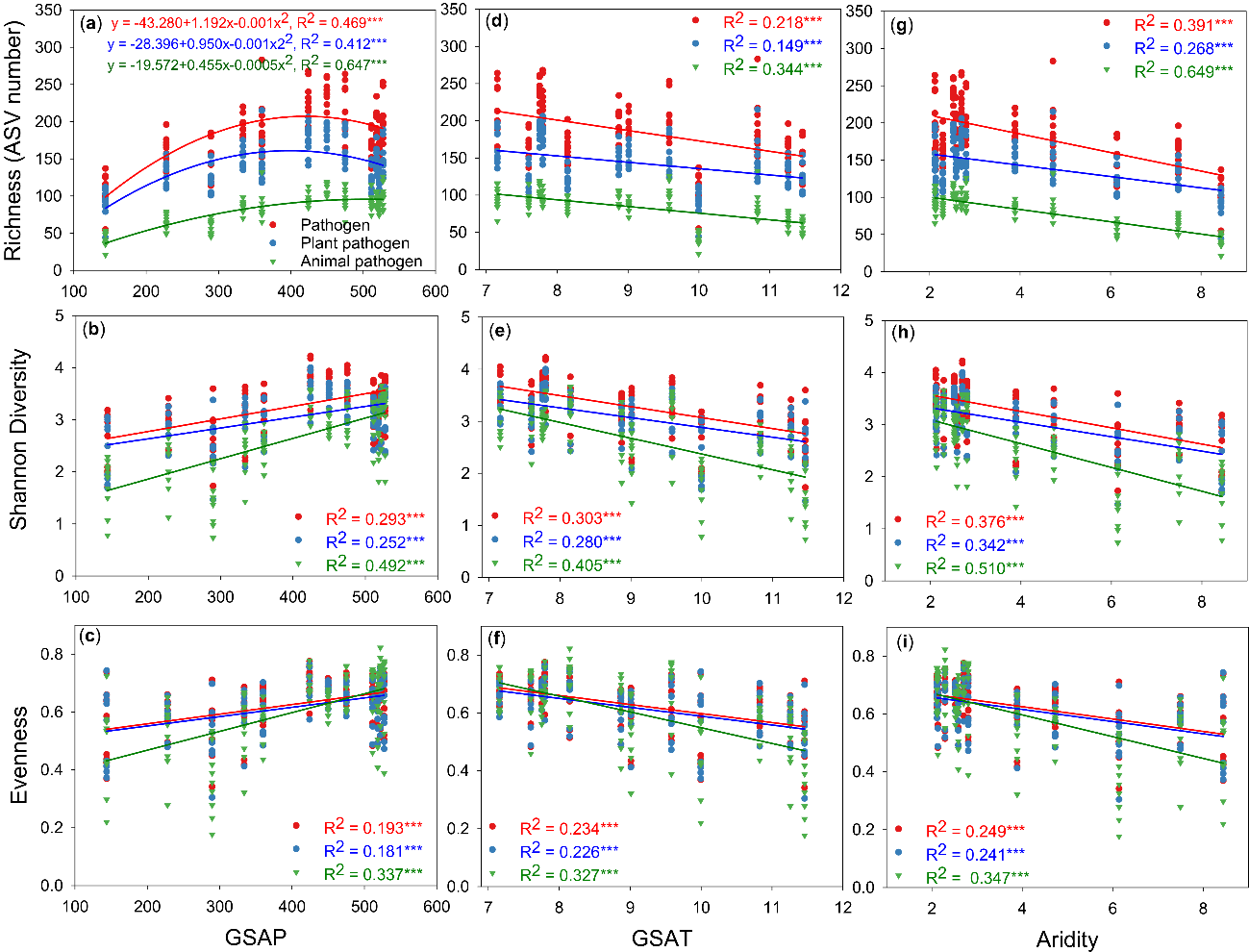


**Figure S11** Correlations between the diversity of various types of pathogens and soil factors. Regressions of pathogen diversity (richness, Shannon diversity, and evenness) with NO_3_^-^ (a-c), NH_4_^+^ (d-f), DN (g-i), DOC (j-l), TOC (m-o) and pH (p-r), including all pathogen diversity, plant pathogen diversity, and animal pathogen diversity. TOC: total organic carbon; DOC: dissolved organic carbon; DN: dissolved nitrogen.


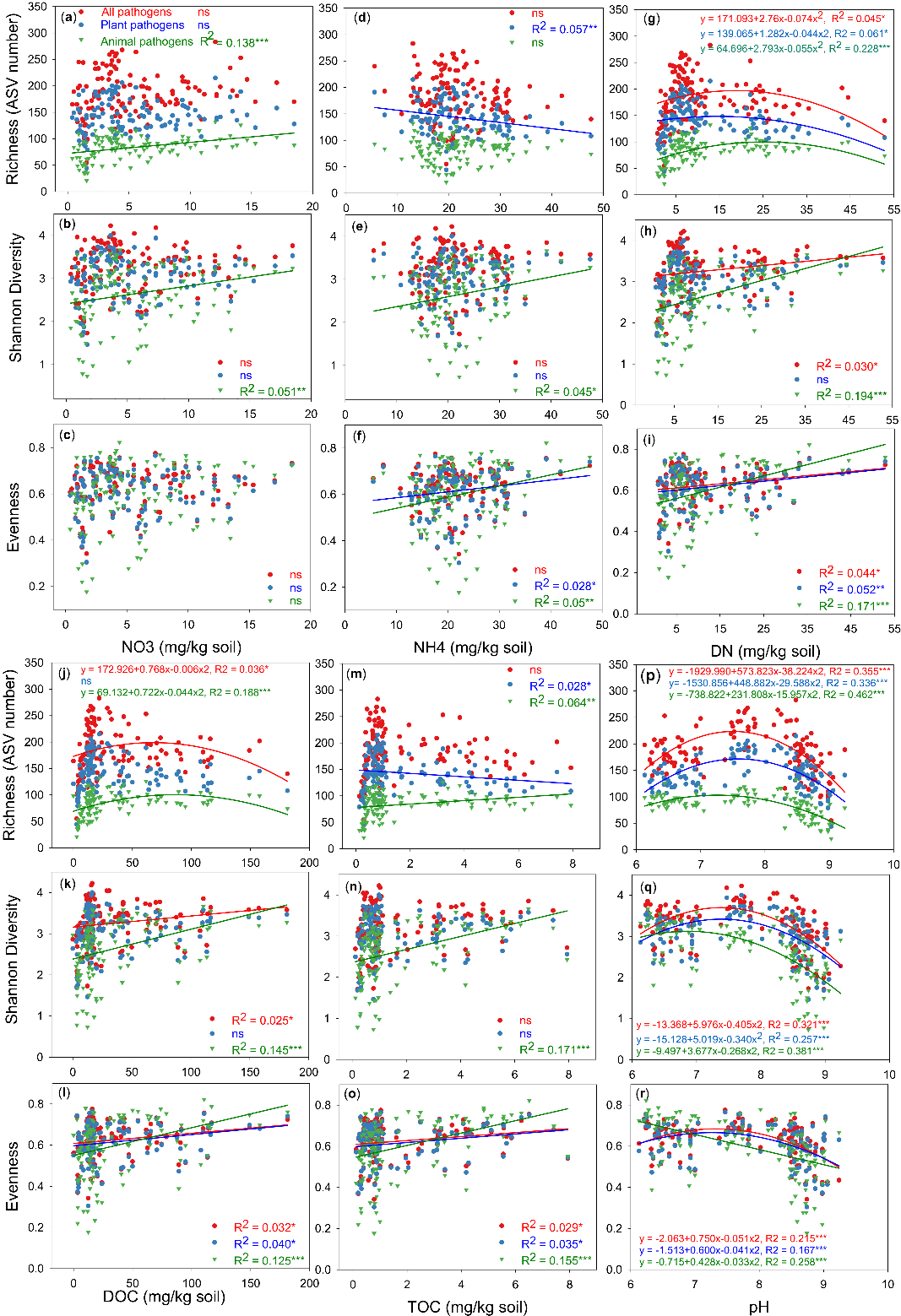


**Figure S12** Correlations between diversity of various types of pathogens and plant diversity. Regressions of pathogen diversity (richness, Shannon diversity, and evenness) with plant diversity(a-c), plant richness (d-f), and plant evenness (g-i), including all pathogen diversity, plant pathogen diversity, and animal pathogen diversity.


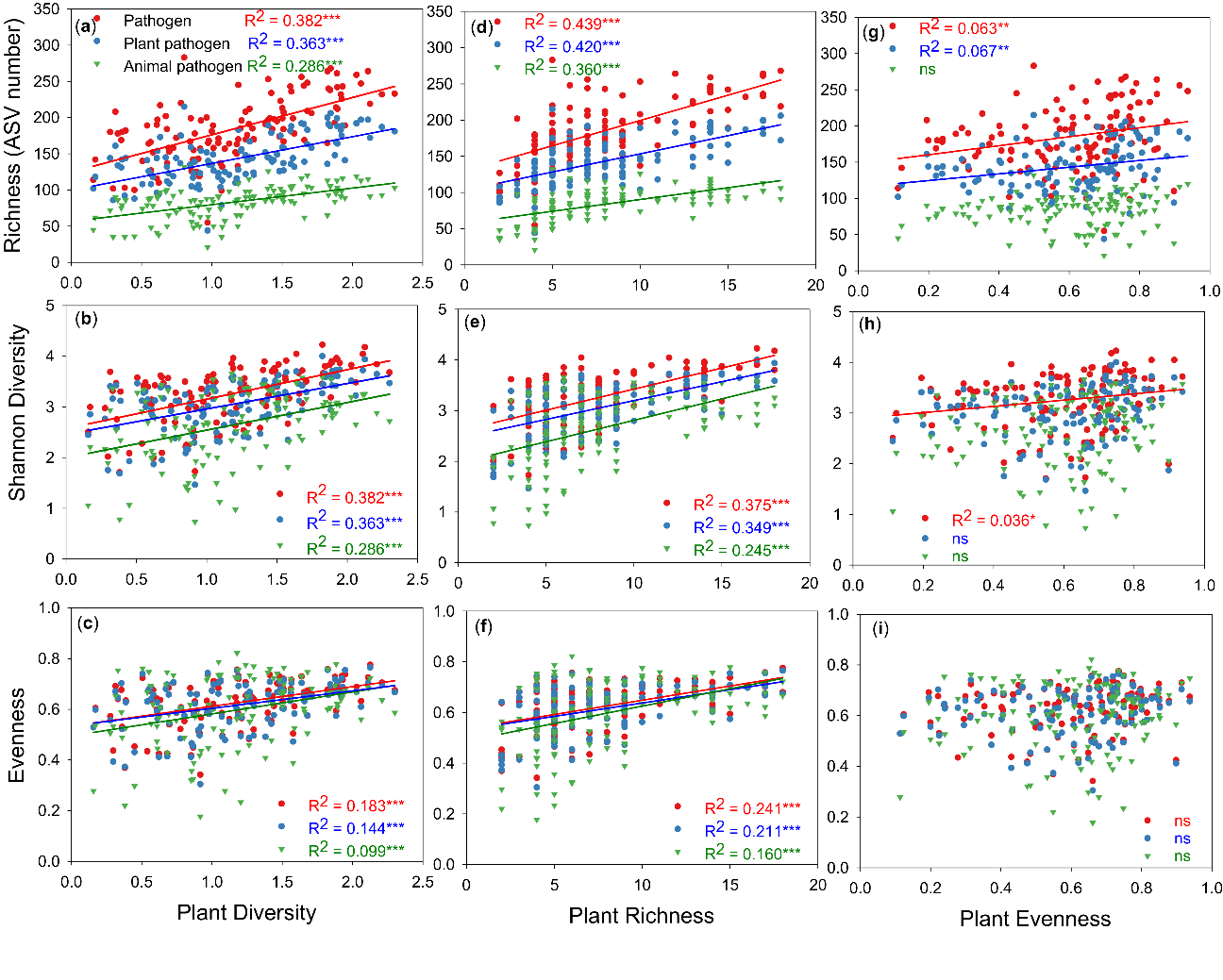


**Figure S13** Correlations between climate factors (precipitation) and soil factors (pH, DOC, DN and TOC). DOC: dissolved organic carbon; DN: dissolved nitrogen; TOC: total organic carbon.

**
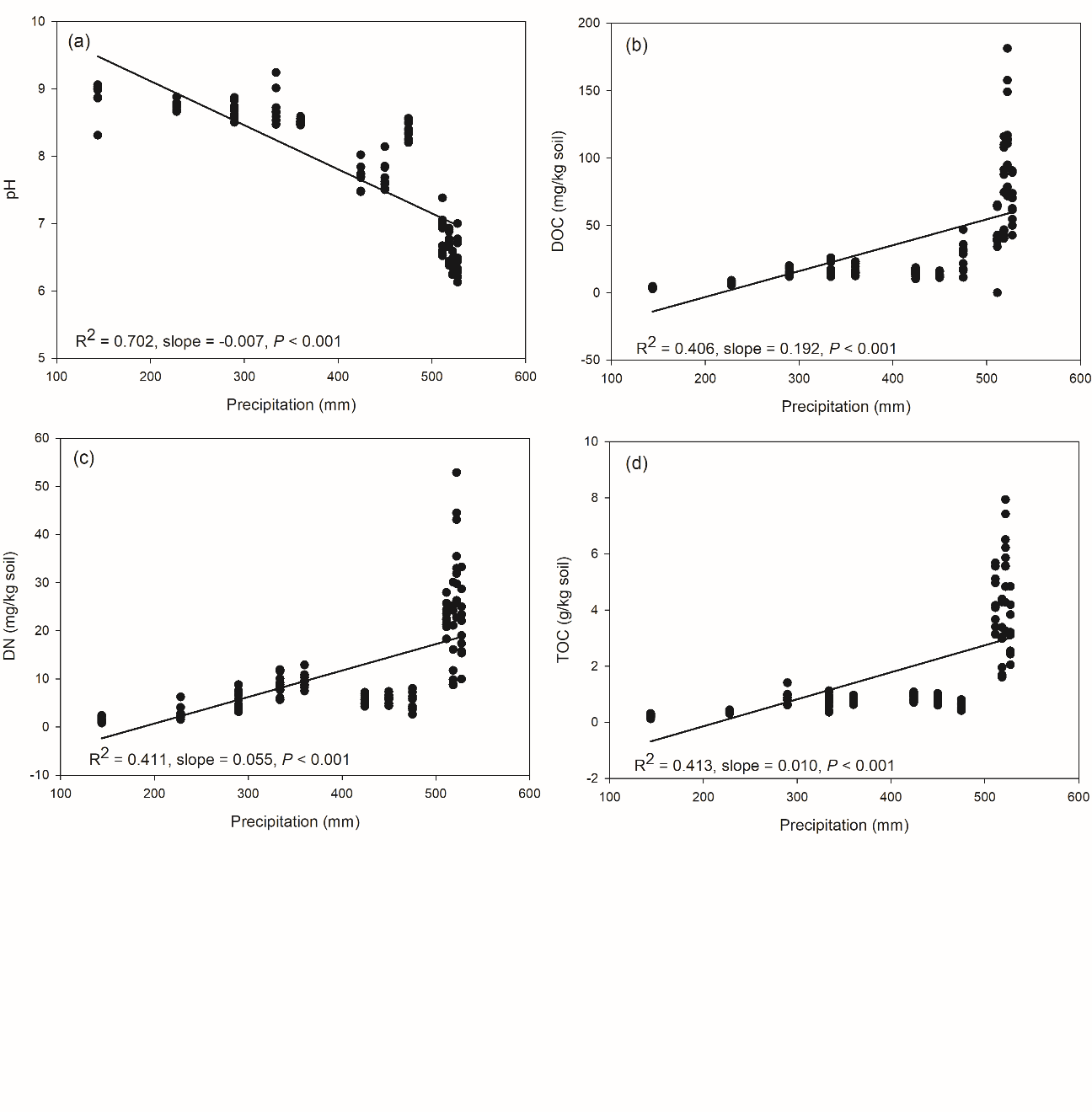
**

**Figure S14** Redundancy analysis (RDA) ordination of pathogen community structure in grazing and fencing grassland showed that environmental factors influencing pathogen community structure were significant (*P* < 0.05). All pathogens (a), plant pathogens (b), and animal pathogens (c) community structure and the driving factors. AP: aboveground plant biomass; PD: plant diversity; PR: plant richness; GSAT: growing season average temperature; GSAP: growing season accumulated precipitation; DOC: dissolved organic carbon; DN: dissolved nitrogen; NH_4_^+^, ammonium-nitrogen; NO_3_^-^, nitrate-nitrogen; TOC: total organic carbon.


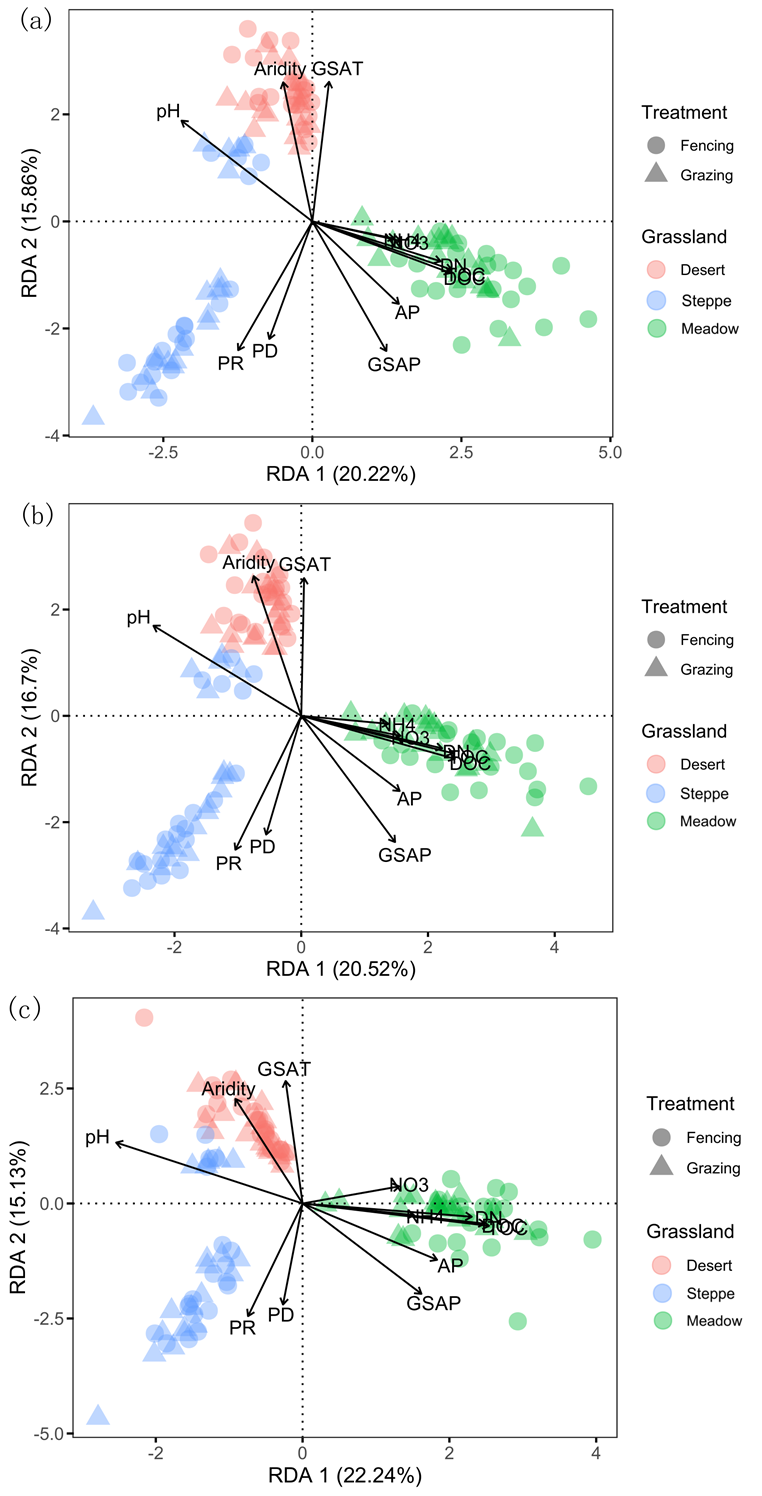


**Figure S15** Variation partitioning analysis of the relative contributions of soil, plant, and climatic variables to variation in non-pathogen community structure. Soil: pH, DOC, DN, NH_4_^+^, NO_3_^-^, TOC; plant: plant diversity, plant richness, aboveground plant-biomass; Climate: aridity degree, growing season average temperature, growing season accumulated precipitation. DOC: dissolved organic carbon; DN: dissolved nitrogen; NH_4_^+^, ammonium; NO_3_^-^, nitrate; TOC: total organic carbon.


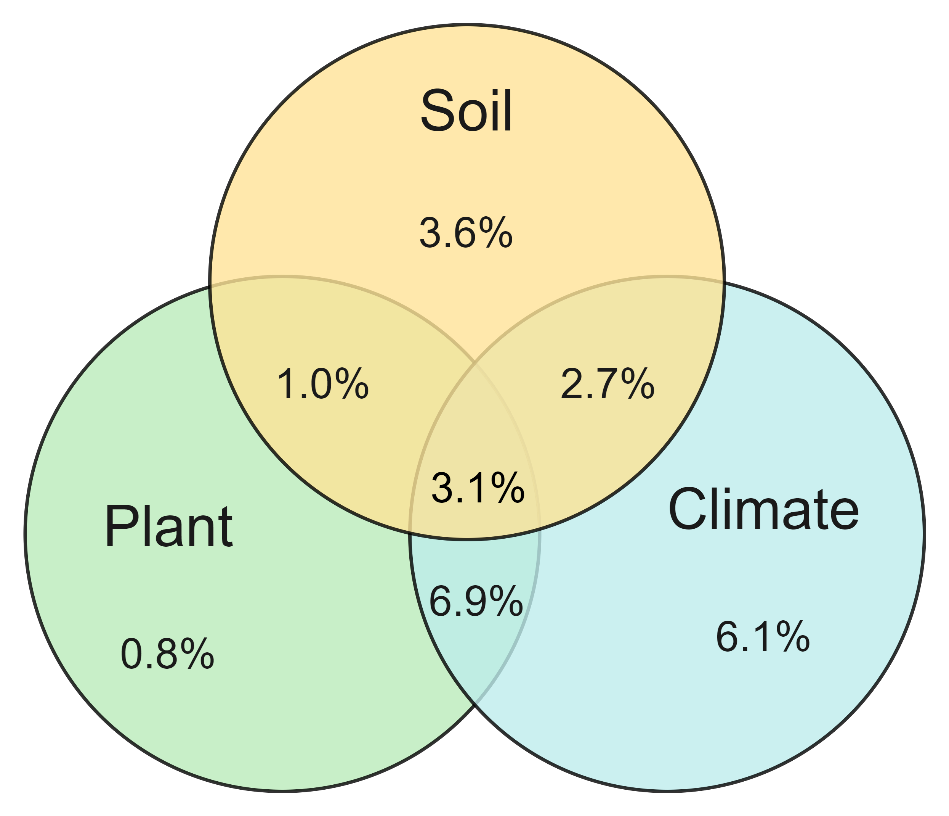


**Figure S16** The pH in desert, steppe, and meadow soils.


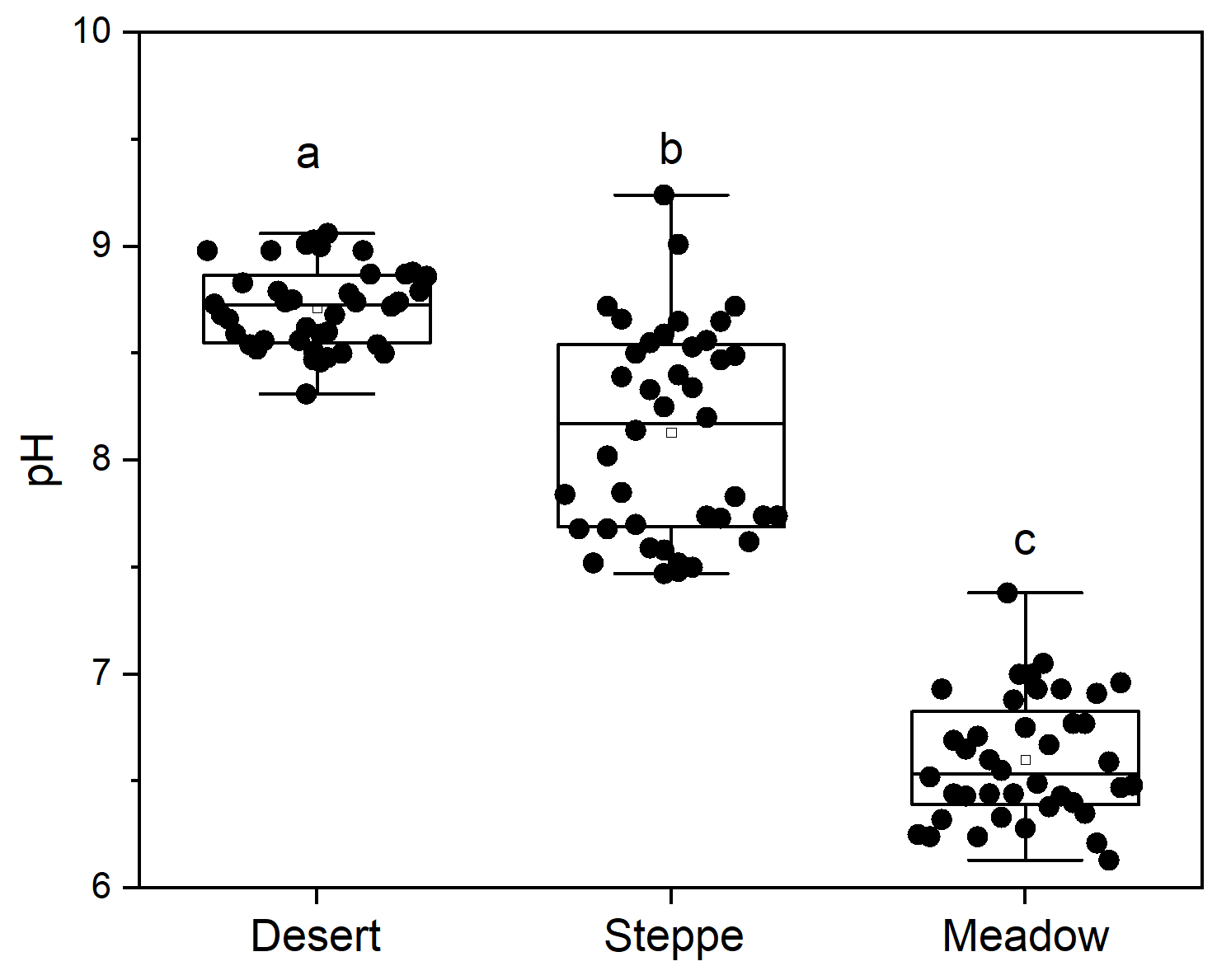


**SUPPLEMENTARY TABLES**

**Table S1.** Estimate and the significance level of richness and Shannon diversity GLM (generalized linear mixed effect model) results for all pathogens (a), plant pathogens (b), and animal pathogens (c).

| Factors | Richness | | Shannon Diversity | |
| --- | --- | --- | --- | --- |
|  | Estimate | *P* value | Estimate | *P* value |
| (a) All pathogens | | | | |
| Grassland | 0.703 | **<0.001** | 0.659 | **<0.001** |
| Grazing | 0.891 | 0.235 | 0.843 | 0.424 |
| Grassland*Grazing | 0.406 | 0.120 | 0.400 | 0.252 |
| (b) Plant pathogens | | | | |
| Grassland | 0.690 | **<0.001** | 0.660 | **0.002** |
| Grazing | 0.200 | 0.200 | 0.852 | 0.671 |
| Grassland*Grazing | 0.104 | 0.104 | 0.401 | 0.451 |
| (c) Animal pathogens | | | | |
| Grassland | 0.653 | **<0.001** | 0.592 | 0.169 |
| Grazing | 0.838 | 0.612 | 0.813 | 0.572 |
| Grassland*Grazing | 0.389 | 0.397 | 0.373 | 0.520 |

*These tests involve predicting the interaction of variables and factors. All samples were tested to include random effects of nested factors within the site. The distribution of the diversity model is Logistic. All significant predictors are in bold.

**Table S2.** Two-way PERMANOVA and Adonis of the pathogen community structure. DF: fenced desert; DG: grazed desert; SF: fenced steppe; SG: grazed steppe; MF: fenced meadow; MG: grazed meadow.

| Subject of samples | All pathogens | |  | Plant pathogens | |  | Animal pathogens | |
| --- | --- | --- | --- | --- | --- | --- | --- | --- |
|  | R^2^ | P |  | R^2^ | P |  | R^2^ | P |
| (a) Two-way PERMANOVA |  |  |  |  |  |  |  |  |
| Grazing | 0.005 | 0.586 |  | 0.005 | 0.574 |  | 0.004 | 0.632 |
| Grassland | 0.321 | **0.001***** |  | 0.339 | **0.001***** |  | 0.362 | **0.001***** |
| Grazing*Grassland | 0.012 | 0.341 |  | 0.011 | 0.387 |  | 0.01 | 0.5 |
| (b) Adonis | |  |  |  |  |  |  |  |
| DF-vs-DG | 0.554 | 0.984 |  | 0.479 | 0.993 |  | 0.49 | 0.971 |
| SF-vs-SG | 1.18 | 0.197 |  | 1.149 | 0.228 |  | 0.926 | 0.516 |
| MF-vs-MG | 1.315 | 0.126 |  | 1.341 | 0.152 |  | 1.182 | 0.228 |

**Table S3.** Explanatory degree of each environmental factor of RDA. AP: aboveground plant biomass; PD: plant diversity; PR: plant richness; GSAT: growing season average temperature; GSAP: growing season accumulated precipitation; TOC: total organic carbon; DOC: dissolved organic carbon; DN: dissolved nitrogen.

| Factors | All pathogens | |  | Plant pathogens | |  | Animal pathogens | |
| --- | --- | --- | --- | --- | --- | --- | --- | --- |
|  | R^2^ | *P* |  | R^2^ | *P* |  | R^2^ | *P* |
| **GSAT** | 0.7014 | **0.001 ***** |  | 0.6853 | **0.001 ***** |  | 0.687 | **0.001 ***** |
| **GSAP** | 0.757 | **0.001 ***** |  | 0.7979 | **0.001 ***** |  | 0.6389 | **0.001 ***** |
| **Aridity** | 0.715 | **0.001 ***** |  | 0.7644 | **0.001 ***** |  | 0.5841 | **0.001 ***** |
| **pH** | 0.8525 | **0.001 ***** |  | 0.8398 | **0.001 ***** |  | 0.8198 | **0.001 ***** |
| **DOC** | 0.6393 | **0.001 ***** |  | 0.6539 | **0.001 ***** |  | 0.6363 | **0.001 ***** |
| **DN** | 0.525 | **0.001 ***** |  | 0.5316 | **0.001 ***** |  | 0.5399 | **0.001 ***** |
| **NH_4_^+^** | 0.2011 | **0.001 ***** |  | 0.1905 | **0.001 ***** |  | 0.2377 | **0.001 ***** |
| **NO_3_^-^** | 0.2351 | **0.001 ***** |  | 0.259 | **0.001 ***** |  | 0.1853 | **0.001 ***** |
| **TOC** | 0.6357 | **0.001 ***** |  | 0.6256 | **0.001 ***** |  | 0.6696 | **0.001 ***** |
| **AP** | 0.4572 | **0.001 ***** |  | 0.447 | **0.001 ***** |  | 0.4841 | **0.001 ***** |
| **PD** | 0.5429 | **0.001 ***** |  | 0.5378 | **0.001 ***** |  | 0.4687 | **0.001 ***** |
| **PR** | 0.743 | **0.001 ***** |  | 0.751 | **0.001 ***** |  | 0.6262 | **0.001 ***** |
